# β‐Mannosyl Triazoles as Mimics of Galactosyl Galectin‐3 and Galectin‐9 N‐Terminal Domain Inhibitors

**DOI:** 10.1002/cbic.70319

**Published:** 2026-04-13

**Authors:** Fredrik Sjövall, Ulf J. Nilsson

**Affiliations:** ^1^ Department of Chemistry Lund University Lund Sweden

**Keywords:** galectin, glycomimetic, mannosyl, Triazole

## Abstract

Mannosyl β‐C‐1 amidotriazoles have previously been reported to have higher selectivity for galectin‐9N (N‐terminal domain) than the corresponding galactoside C3 amidotriazoles have, which were more selective for galectin‐3. This study further investigated this by synthesis of mono‐ and bis‐aryltriazolyl mannoside analogues to known high‐affinity galectin‐3 galactosyl‐derived inhibitors. Following synthesis, affinity measurements using competititve fluorescence polarization assays were performed which indicated low affinity of the bis‐aryltriazolyl mannosyls, while the mono‐aryltriazolyl mannosyls analogs possessed improved affinity, albeit with lower and selectivity. From conformational calculations it was implied that the weak‐binding bis‐aryltriazolyl mannosyl analogues do not find the same conformation and binding pose as the parent galactoside compounds.

## Graphical abstract

Galacto‐mimicking mannosyl monotriazoles, but not bis‐triazoles, inhibit galectin‐3 and galectin‐9 N‐terminal domain.

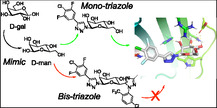

## 1 Introduction

The family of proteins known as galectins are a group of small, soluble proteins found in most human tissues [1]. A key property of galectins is the high affinity for β‐D‐galactosides present as a common monosaccharide unit in glycoconjugates such as glycoproteins and glycolipids [2]. A binding site of glycan‐binding proteins is referred to as the carbohydrate recognition domain, CRD [2], which, in case of galectins, binds β‐D‐galactoside‐carrying glycoconjugates. Galectins bind glycoconjugates and play roles in cellular communication [1], and they are linked to immunoregulation, tumor growth, and inflammation [3]. Galectin‐9 is a tandem‐repeat type protein, with two different CRDs, the N‐and the C‐terminal [4]. It has been identified to bind to TIM3 and PD‐1, regulatory proteins found on T‐cells [5], to down‐regulate T‐cell activities [4, 5, 6, 7, 8, 9, 10, 11]. Hence, inhibition of galectin‐9 could improve treatment of immunological disorders and cancer [11, 12, 13, 14, 15, 16].

### 1.1 Mannosyls as Galactosyl Mimics

**FIGURE 1:**
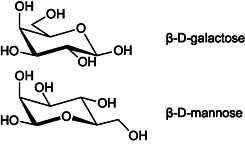
β‐D‐Galactose (above) and β‐D‐mannose (below).

**FIGURE 2:**
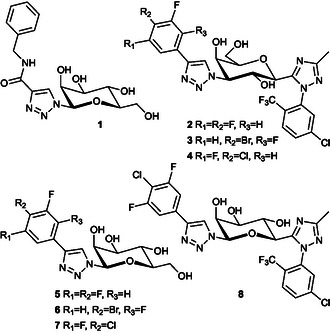
Known mannosyl triazole **1** and galactoside **2–4** galectin‐inhibitors. The mannosyl‐derived bis‐triazolyl galectin‐inhibitors **5–8** (this work).

Tejler et al. [17] found that β‐mannosyl amidotriazoles bind to the CRD of galactose‐recognizing galectin‐3 and 9N, but not to galectin‐1, 7, or 8N. The mannosyl triazoles were selective for galectin‐9N over galectin‐3 compared to their galactosyl analogs, with sub‐mM affinity for galectin‐9N. Interactions of the mannosyls with the galectin‐3 and 9N were explained with mannose serving as a galactose mimic, i.e., the axial mannosyl 2OH mimicking the axial galactosyl 4OH (Figure 1). The most potent inhibitor (**1**, Figure 2) had a K_d_ value of 540 μM for galectin‐9N and 1900 μM for galectin‐3. Furthermore, the mannosyl amidotriazoles were described as easier to synthesize compared to the corresponding C3 amidotriazolyl galactosides.

In a recent publication, Liu et al. high‐affinity bis‐triazolyl galactoside‐derived galectin‐3 inhibitors, such as **2–4** [18], carrying C3 halophenyl‐1H‐1,2,3‐triazolyl and C1 phenyl‐1,2,4‐triazolyl rings were reported for galectin‐3. The 1,3‐bis‐triazolyl‐galactosyl inhibitors **2–4** (Chart 1) display low nanomolar IC_50_ values for galectin‐3, while being significantly worse for galectin‐9 in an ELISA assay. Installing the 1,2,4‐triazolyl moieties of **2**‐**4** on a galactosyl scaffold requires adding an exocyclic carbon onto the C1 before performing the cyclization. This exocyclic C1 carbon will correspond to mannosyl C6 when using mannose as a galacto‐mimicking scaffold (Figure 1). Thus, mimicking the galactosyls **2**‐**4** with mannose‐scaffolded counterparts carrying a C1 halophenyl‐1H‐1,2,3‐triazolyl and a C6 phenyl‐1,2,4‐triazolyl moiety could allow for shorter syntheses that do not require installation of an extra exo‐cyclic carbon. Hence, we hypothesized that replacing the galactopyranosyl ring in **2**‐**4** with the galactose‐mimicking mannopyranosyl ring (as in **1**) would yield mannosyl mono‐triazoles **5**‐**7** as potential galectin‐3 and 9N inhibitors and that incorporation of a 1,2,4‐triazole at mannose C6 would provide a 1,6‐bis‐triazolyl‐mannosyl derivative **8** carrying two affinity‐enhancing triazole moieties as in **2**‐**4** hypothesized to enhance affinity for galectin‐3. Herein, we disclose the synthesis and evaluation of mannosyl analogs **5**‐**8** as galectin inhibitors, as well as of a 4‐deoxy **21** and 4,5‐olefinic **23** analog of **8**.

## 2 Results and Discussion

### 2.1 Synthesis and Galectin Affinity Evaluations

**SCHEME 1:**
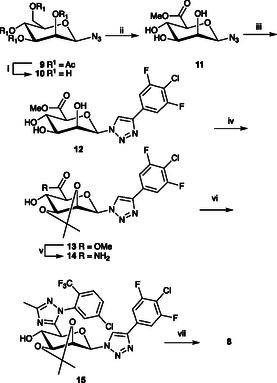
Reagents and conditions: (i) NaOMe/MeOH, 20 min, rt, quant. (ii) a) NaOCl (13%), NaHCO_3_ (sat. aq. sol.), NaBr, TEMPO, H_2_O, 0°C, 18 h, b) DOWEX 50W, MeOH, rt, 18 h. 64% over two steps. (iii) 2,2‐Dimethoxypropane, CSA, rt, 18 h, 82%, (iv) ((4‐chloro‐3,5‐difluorophenyl)ethynyl)trimethylsilane, CuI, Et_3_N, ACN, N_2_, rt, 18 h, 51%, (v) NH_3_ (7M in MeOH), 4 h, rt, quant. (vi) a) DMA–DMA, 1,4‐dioxane, 80°C, 2 h, b) 2‐chloro‐5‐(trifluoromethyl)‐phenyl‐hydrazine, AcOH, 80°C 1h, (vii) AcOH:H_2_O, 80°C, 72 h, 7% over three steps.

The starting material for compounds **5–8** was the mannosyl azide **9**, which was synthesised according to established literature protocols [19], except that DMF was used as the solvent. First, the monofunctionalized triazolyl β‐mannosyl derivatives **5–7** were synthesized via CuAAC reactions of the azide **9**, followed by deprotection under Zemplén conditions. Synthesis of a bis‐triazole **8** analog to the chloro‐difluoro compound **7** started with deprotection of **9** under Zemplén conditions to give **10** (Scheme 1). Selective TEMPO‐mediated oxidation of 6OH of **10** with NaOCl as co‐oxidant and NaBr as co‐catalyst gave an intermediate mannuronic acid derivative, which was highly polar and proved difficult to purify with flash chromatography. Instead, the crude acid was concentrated and subjected to Fischer esterification conditions, which afforded the methyl ester **11**. Then, **11** was reacted with ((4‐chloro‐3,5‐difluorophenyl)ethynyl)trimethylsilane under CuAAC conditions to give the triazole **12**. Isopropylidene‐protection of **12** gave the 2,3‐acetal **13**, which was aminolysed to afford the primary amide **14**. The amide **14** was, via its DMA–DMA intermediate, cyclized [20] at 80°C to the triazole **15**. Isopropylidene removal of **15** gave the target bis‐triazole **8**.

**SCHEME 2:**
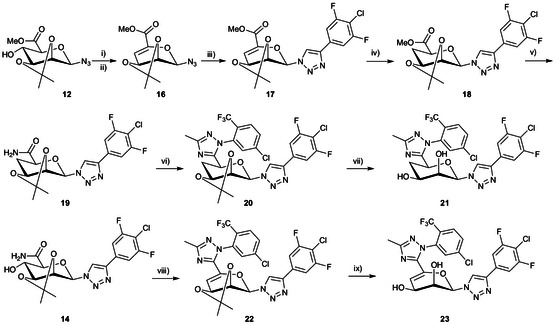
Reagents and conditions: (i) MsCl, py, rt, 18 h, 68%, (ii) Al_2_O_3_, py, 40°C, 18 h, quant. (iii) ((4‐chloro‐3,5‐difluorophenyl)ethynyl)trimethylsilane, CuI, Et_3_N, ACN, N_2_, rt, 72 h, 75%, (iv) EtOH, H_2_, Pd/C, rt, overnight, quant. (v) NH_3_/MeOH, MeOH, rt, 18 h, quant. (vi) a) DMA–DMA, 1,4‐dioxane, reflux, 24 h and b) 2‐chloro‐5‐(trifluoromethyl)‐phenyl‐hydrazine, AcOH, 80°C 1h, (vii) AcOH:H_2_O, 80°C, 72 h, 8% over three steps, viii) DMA–DMA, 1,4‐dioxane, reflux, 18 h then 2‐chloro‐5‐(trifluoromethyl)‐phenyl‐hydrazine, AcOH, 80°C 1h, (ix) AcOH:H_2_O, 80°C, 72 h, 2% over three steps.

Evaluation of **5**‐**8** as galectin inhibitors in competitive fluorescence polarization assays indicated mid‐micromolar affinity of the mono‐triazoles **6‐7**, weaker mM affinity of **5**, and an almost non‐inhibitory effect of the bis‐triazole **8** (Table 1)**.** While the trifluoro derivative **5** displays an affinity for galectin‐3 similar to that of the amide **1**, the bromo and chloro mono‐triazoles **6**‐**7** represent an improvement over **1** as galectin‐3 inhibitors with affinities approaching that of lactose (K_d_ 220 µM [21]) and significantly better than that of methyl galactoside (K_d_ 4400 µM [21]). In case of galectin‐9N, the trifluoro derivative **5** loses affinity compared to the amide **1**, while the bromo and chloro compounds **6**‐**7** are again better than **5** and similar to **1**. Hence, the mannosyls **6** and **7** may serve as novel scaffolds devoid of a polar and metabolically susceptible amide moiety in discovery efforts toward galectin‐3 and ‐9N inhibition. The barely detectable binding by the 1,6‐derivatized mannoside **8** to galectin‐3 remains a disappointing observation, while the loss of binding to galectin‐9N is expected, given that the parent galactoside **4** has been reported to be a significantly poorer inhibitor of galectin‐9 than of galectin‐3 [18]. One reason for the loss of affinity of **8** for galectin‐3 compared to **4** could, at least in part, be that **8** adopts a galactose‐mimicking pose when binding to a galectin, a galactosyl ligand O5 hydrogen bond is replaced by the mannosyl C4‐OH, which cannot efficiently mimic a galactosyl O5 key hydrogen bond to a conserved arginine side chain. In addition, a consequence of either steric hindrance and/or electronic mismatch by the mannosyl 4OH can possibly be that this prevents a C6 *N*‐phenyl‐1,2,4‐triazole conformation required for an efficient backbone–halogen bond (as observed for the corresponding galactose derivative **4** [18] (pbd ID 7XFA). Hence, the 2OH in galactosyls and 4OH in mannosyls presumably affect the conformation of the 1‐(5‐chloro‐2‐trifluoromethylphenyl)‐1,2,4‐triazole differently in such a manner that its conformation in a mannosyl derivative cannot engage as efficiently in a critical 5‐chloro‐mediated halogen bond to a conserved glycine backbone carbonyl oxygen [18]. The X‐ray (pdb ID 7XFA) of galectin‐3 in complex with **4** reveals that the 2O of **4** is in near hydrogen bond distance to the 1,2,4‐triazole N4 and to a fluorine atom of the trifluoromethyl group (3.3 Å in both cases), which suggests that the 2OH of **4** may be important for a 1‐(5‐chloro‐2‐trifluoromethylphenyl)‐1,2,4‐triazole halogen bonding conformation. Another reason may be that the presence of a mannosyl 4OH also clashes with and tilts the 1,2,4‐triazole ring in **8** toward O5 and thus induces an altered conformation of the 1‐(5‐chloro‐2‐trifluoromethylphenyl)‐1,2,4‐triazole that is unable to form an efficient halogen bond with the backbone Gly83 carbonyl oxygen. In order to test this hypothesis, we synthesized the 4‐deoxy analog **21** of the mannosyl **8** (Scheme 2), as the 4‐deoxy methylene in **21** would be sterically similar to the ring O5 in the parent galactosyl **4**, thus avoiding any steric clash by a mannosyl 4OH.

**TABLE 1:** Dissociation constants (µM) for mannosyls **1** and **5**–**8** for galectin3 and 9N determined in a competitive fluorescence polarization assay.

	Galectin‐3	Galectin‐9N
**1** [17]	1900	540
**5**	1500 ± 230	2100 ± 360
**6**	290 ± 30	440 ± 10
**7**	240 ± 50	740 ± 30
**8**	≫2400^a^	≫2400

a Non‐inhibitory at the highest tested concentration 2400 µM.

Attempts with a Barton–McCombie deoxygenation of **12** via formation of a xanthate or thionoester failed and instead gave the Chugaev‐elimination product **16**. However, as this serendipitously formed an unsaturated deoxygenation product **16**, it was chosen to proceed with compound **16** and to saturate it. Compound **16** was also readily obtained via mesylation of 4OH, followed by elimination upon heating under alkaline conditions. Prior to reduction, compound **16** was converted to the phenyltriazolyl **17** to avoid simultaneous reduction of the azide. Catalytic hydrogenation over Pd/C afforded the desired 4‐deoxy derivative **18** with exclusive hydrogen addition to the mannopyranosyl β‐face, as confirmed by NOESY experiments. Aminolysis of **18** gave **19** and cyclization of **19** towards the triazole **20** was attempted**.** Formation of the reactive intermediate required for cyclization proved sluggish but could be achieved by increasing the temperature to reflux. Cyclization to **20** was successful and subsequent deprotection afforded **21**. Inhibitors derived from D‐galactal were recently reported to display increased affinity for galectin‐8N, which was proposed to be a result of an overlap of the D‐galactal olefin HOMO with the galectin‐8N Arg45 LUMO [22]. Hence, it was of interest to proceed and install the C6 1,2,4‐triazole moiety on the olefinic **17** and evaluate it as inhibitor of galectin‐9N or galectin‐3. Galectin‐3 and 9N CRDs present arginine side chains, Arg65 and Arg77, respectively, near a bound galactose O5‐C1, which would correspond to mannose C4‐C5. Refluxing the amide **14** with DMA–DMA to form the imide cyclization precursor was accompanied by 4OH elimination and subsequent cyclization afforded the unsaturated analog **22.** Isopropylidene hydrolysis gave alkene **23**.

Evaluation of **21** as inhibitor of galectin‐3 and 9N revealed that deoxygenation does not increase in affinity (Table 2) and thus steric influence by the 4‐OH on the 1,2,4‐triazole of the parent mannosyl **8** does not seem to be a major reason for the loss of affinity of **8** compared to **4**. The 4,5‐alkene **23** did not show any inhibition of galectin‐3 or galectin‐9N at the concentrations tested, which suggests that the alkene in **23** likely does not engage in any affinity‐enhancing π‐interactions, as hypothesized above.

**TABLE 2:** Dissociation constants (µM) for mannosyls **21** and **23** for galectin‐3 and 9N determined in a competitive fluorescence polarization assay.

	Galectin‐3	Galectin‐9N
**21**	≫2400^a^	≫2400
**23**	≫2400	≫2400

a Non‐inhibitory at the highest tested concentration 2400 µM.

### 2.2 Molecular Dynamic Simulations

**FIGURE 3:**
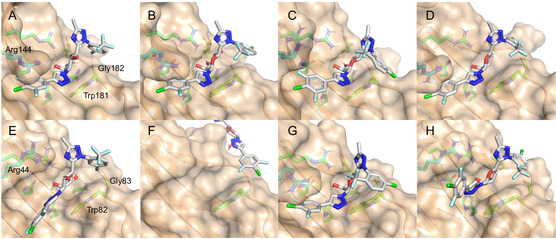
Binding poses with galectin‐3 of the (A) galactosyl **4** (X‐ray, pdb id 7XFA) and the mannosyl derivatives (B) **8**, (C) **21**, and (D) **23**. Binding poses with galectin‐9N of the (E) galactosyl **4** and the mannosyl derivatives (F) **8**, (G) **21**, and (H) **23**. The complexes (B–H) are from Desmond cluster analyses of resulting trajectories from molecular dynamics simulations, where the images show the most prevalent ligand conformations. The MD of mannosyl derivative **8** in complex with galectin‐9N (F) did not result in a stable pose and an arbitrary out‐of‐site ligand pose is shown. Hydrogen bonds to the pyranose moieties are shown with yellow dashed lines and halogen bonds to the Gly182/Gly83 carbonyl oxygen are shown with magenta dashed lines.

To further investigate the binding modes of the mannosyl galectin inhibitors, molecular dynamics simulations of **7**‐**8**, **21**, and **23** in complex with galectin‐3 and ‐9N were performed. The published crystal structure 7XFA with galactosyl **4** bound to galectin‐3 was imported and prepared in Schrödinger's Maestro (Figure 3A). The galactosyl **4** was converted to mannosyls **7**‐**8**, deoxy‐mannosyl **21**, and the mannosyl olefin **23**. Trajectory cluster analyses of the MD simulations of the five complexes of **4**, **7**‐**8**, **21**, and **23** with galectin‐3 showed that the mono triazolyl mannosyl **7** finds a pose mimicking that of **4** with a virtually similar phenyl‐triazolyl stacking with Arg144. However, the mannosyl derivatives **8**, **21,** and **23** adopt different bound conformations (Figure 3B–D) compared to the galactosyl **4**. Nevertheless, the 4‐chloro‐3,5‐difluorophenyl triazoles of **8** and **23** present interaction with the Arg144 side chain analogous to that observed in the crystal structure of **4** bound by galectin‐3, which possibly explains why the mannosyls **5**‐**7** display good affinities for galectin‐3. However, altered binding conformations of **8**, **21**, and **23** lead to loss of ligand‐protein binding interactions: the hydrogen bond network with conserved amino acid side chains and the Gly182 halogen bond seen for galectin‐3‐bound **4** are lost (Figure 3). The galactosyl 6OH of **4** fills a small pocket to participate in a network of hydrogen bonds with the surrounding residues. Mannosyl 3OH in **8**, mimicking the galactosyl 5CH_2_OH in **4**, does not extend deep enough into this pocket to properly mimic the hydrogen bond network observed in the X‐ray structure of **4** in complex with galectin‐3, which, at least partly, explains the weaker affinity of mannosyls **8**, **21**, and **23**. Furthermore, the mannosyl 4OH in **8** is likely a steric blocker for the phenyl‐1,2,4‐triazolyl moiety to adopt a conformation allowing for formation of the critical halogen bond to Gly182 (as formed with the galactosyl derivative **4**).

An analogous MD analysis was performed for galectin‐9N. Galectin‐9N in complex with **4** was built from the X‐ray structure of galectin‐9N in complex with a seleno‐lactose derivative (pdb id 3WLU [23]). Briefly, the pyran ring of **4**, with the **4** substituents and functional groups kept in the conformations determined in the galectin‐3:**4** X‐ray structure (7FXA), was overlayed with the galactopyranose ring of the selenolactose derivative in 3WLU to generate a starting conformation for MD analysis. As described for galectin‐3 above, the galectin‐9N complexes with the mannosyls **8**, **21**, and **23** were in turn built from the complex with **4**. Trajectory cluster analysis of the galectin‐9N complex with **4** revealed that the galactopyranosyl and the anomeric phenyl‐triazole moieties retained the conformation and protein interactions as observed in the X‐ray structure of the corresponding galectin‐3 complex (Figure 3E). Hence, the galactosyl 4‐ and 6OH hydrogen bonds in the conserved galactose site and the halogen bond between the anomeric chlorophenyl and galectin‐9N Gly83 were present. The galactose C3 phenyl‐triazole moiety preferred a conformation different from that of the galectin‐3 complex and sampled different and less persistent interaction geometries with the Arg44 side chain in galectin‐9N. The lack of a distinct phenyl–triazole interaction with an arginine side chain in galectin‐9N may, at least partly, explain why **4** is a less potent inhibitor of galectin‐9 than of galectin‐3 [18]. The simulations of the complex between **7** and galectin‐9N revealed a galacto‐mimicking pose of **7** similar to the result of the simulation of the galectin‐3 complex. Hence, also for galectin‐9N the comparatively good affinity of the mannosyl triazoles **5**‐**7** may be attributed to mimicry of C3‐triazolyl galactosides. The mannosyl derivative **8** failed to find a stable pose bound to galectin‐9N during the MD simulation (Figure 3F shows an arbitrary selected view with **8** becoming unbound). In contrast, the deoxy **21** and olefinic **23** analogs remained bound to galectin‐9N during the MD simulations and notably formed frequent interactions with the galectin‐9N Arg44 side chain, but with their chlorophenyl‐1,2,4‐triazolyl moieties unable to engage in halogen bonding with Gly83 (Figure 3G–H). A pronounced interaction of a mannosyl C1 halo‐phenyl‐triazole, as in **21** and **23,** with the Arg44 side chain may, at least in part, explain the observed affinity of the mono‐triazoles **6**‐**7** for galectin‐9N.

Hence, while the mannosyls **8**, **21**, and **23** according to the MD analyses do mimic galactose binding and interactions with galectin‐3 and ‐9N (except for **8** bound to galectin‐9N), their much weaker affinities may be explained by that they lack the galactopyranose ring O‐5 hydrogen bond with an arginine side chain, the mannosyl 3‐OH forms less efficient hydrogen bonding compared to the galactosyl 5CH_2_OH, and they do not adopt bound conformations engaging their chlorophenyl‐1,2,4‐triazolyl bi‐aryls in halogen bonding with glycine backbone carbonyl oxygens.

## 3 Conclusions

While the mono‐triazolyl mannosyls **5**‐**7** indeed proved to be inhibitors of galectin‐3 and galectin‐9N with affinities improved over known mannosyl‐based inhibitors [17], the 1,6‐difunctionalized mannosyl derivatives **8**, **21**, and **23** did not offer any enhanced affinities. Taken together, the study delivers novel fundamental insight in the mannose‐galactose mimicry and in glycomimetic ligand–galectin interaction. Mannosyls may be exploited as galactosyl mimics, but derivatization should consider that structural differences between galactose and mannose may influence how a particular derivatization affects inhibitor conformation and thus protein interactions. Here, installing a phenyl‐1,2,4‐triazolyl moiety at mannopyranosyl C6 does not improve affinity likely a consequence of a mannose 2OH influencing the conformation of the 1,2,4‐triazole moiety differently from that of the ring O5 in the parent galactosyl structure.

## 4 Experimental

### 4.1 General

All reagents and solvents were dried before use according to standard methods. Commercial reagents were used without further purification. TLC analysis was performed on precoated Merck silica gel 60 F254 plates using UV light and charring solution (H_2_SO_4_: EtOH 1:9). Flash column chromatography was performed on SiO_2_ purchased from Aldrich (technical grade, 60 Å pore size, 230–400 mesh, 40–63 μm) or using Biotage Sfär Silica HC D cartridges on a Biotage Isolera System. Preparative HPLC was performed on an Agilent 1260 Infinity system with a SymmetryPrep C18, 5 μM, 19 mm × 100 mm column using a gradient (water with 0.1% formic acid and acetonitrile). Monitoring and collection were based on UV–vis absorbance at 210 and 254 nm. NMR spectra ^1^H, ^13^C, 2D COSY were recorded with a Bruker Avance II 400 MHz spectrometer (400 Hz for ^1^H, 100 Hz for ^13^C) or a Bruker Avance III 500 MHz spectrometer (500 Hz for ^1^H, 125 Hz for ^13^C) at ambient temperature. Chemical shifts are reported in δ parts per million (ppm), with multiplicity (b = broad, s = singlet, d = doublet, t = triplet, q = quartet, quin = quintet, hept = heptet, m = multiplet, app = apparent), coupling constants (in Hz), and integration. High‐resolution mass analyses were performed using a Micromass Q‐TOF mass spectrometer (ESI). Purities of final compounds were determined by UPLC (Waters Acquity UPLC system, column Waters Acquity CSHC18, 0.5 mL min^−^^1^ H_2_O–MeCN gradient 5%–95% 10 min with 0.1% formic acid). Analytical data are given if the compound is novel or not fully characterized in the literature. All tested compounds were ≥95% pure according to analytical HPLC analysis. Fluorescence polarization experiments were performed as described [24, 25] with the specific conditions as reported [26].

### 4.2 1‐[4‐(3,4,5‐Trifluorophenyl)‐1H‐(1,2,3)‐Triazol‐1‐Yl]‐1‐Deoxy‐β‐D‐Mannopyranose 5

In a round bottom flask containing compound **9** (1 g, 2.68 mmol), ACN (20 mL), CuI (102 mg, 0.54 mmol, 0.2 eq.), Et_3_N (0.7 mL, 5.36 mmol, 2 eq.), and 3,4,5‐trifluorophenylacetylene (0.36 mL, 2.95 mmol, 1.1 eq.) were added. The solution was put under a N_2_ atmosphere and set to stir at room temperature for 18 h after which TLC indicated full conversion. The reaction was stopped by concentrating the crude and adding saturated aqueous NH_4_Cl (50 mL), which was then extracted with DCM (4 × 30 mL), and the combined organic layers were collected and dried using MgSO_4_ before removing the solvent in vacuo. This left a white foamy crude with some yellow impurity which solidified to an amorphous white solid (1.41 g, quant.).

To the crude, MeOH (25 mL) was added along with methanolic NaOMe (1 M, 5 mL). The dispersion was set to stir at rt for 1 h, after which TLC and LC‐MS indicated full conversion. The mixture was neutralized with DOWEX 50W, which was then filtered off. The filtrate was concentrated under reduced pressure, and the solid residue purified using column chromatography (9:1 DCM:MeOH), which afforded **5** as a white solid (920 mg, quant.).

1H NMR (400 MHz, MeOD) δ 8.66 (s, 1H, triazole‐H), 7.70–7.57 (m, 2H, Ar‐H), 6.08 (d, *J* = 1.3 Hz, 1H, H‐1), 4.19 (t, *J* = 1.9 Hz, 1H, H‐2), 3.98 (dd, *J* = 12.1, 2.3 Hz, 1H, H‐6), 3.87–3.74 (m, 3H, H‐3, H‐4, H‐6), 3.59 (dqd, *J* = 10.0, 6.0, 2.2 Hz, 1H, H‐5).

^13^C NMR (101 MHz, MeOD) δ 151.48 (ddd, *J* = 247.9, 10.1, 4.1 Hz), 143.94, 139.15 (dt, *J* = 250.4, 15.1 Hz), 127.52–127.12 (m), 121.58, 109.62–109.06 (m), 87.04, 80.08, 73.49, 70.83, 66.34, 61.23.

HRMS calculated for [C_14_H_15_F_3_N_3_O_5_]^+^ (M + H^+^): 362.09638, found 362.0959.

### 4.3 1‐[4‐(4‐Bromo‐2,3‐Difluorophenyl)‐1H‐(1,2,3)‐Triazol‐1‐Yl]‐1‐Deoxy‐β‐D‐Mannopyranose 6

Starting material **9** (70 mg, 0.19 mmol) was added to a round‐bottom flask, and dissolved in ACN (5 mL) put under a N_2_ atmosphere. To the solution, CuI (7 mg, 0.04 mmol, 0.2 eq.), Et_3_N (52 µL, 0.37 mmol, 2 eq.) and ((4‐bromo‐2,3‐difluorophenyl)ethynyl)trimethylsilane (54 mg, 0.19 mmol, 1 eq.) were added and the solution was set to stir at rt for 18 hr after which TLC and LCMS indicated full conversion of the starting material. The reaction mixture was concentrated, and the crude material was dissolved in MeOH (4 mL) to which a solution of NaOMe in MeOH (1 M, 1 mL) was added. After 1 h, TLC and LCMS indicated full conversion of the intermediate and the reaction mixture was neutralized using DOWEX 50W. The resin was filtered off and the filtrate was collected and concentrated before purifying using prep‐HPLC which afforded **6** as a white amorphous solid after lyophilization (17 mg, 0.04 mmol, 21%).

^1^H NMR (500 MHz, MeOD) δ 8.65 (d, *J* = 3.6 Hz, 1H, triazole‐H), 7.89 (ddd, *J* = 8.9, 6.9, 2.1 Hz, 1H, Ar‐H), 7.56 (ddd, *J* = 8.5, 6.3, 2.0 Hz, 1H, Ar‐H), 6.14 (d, *J* = 1.3 Hz, 1H, H‐1), 4.18 (t, *J* = 1.8 Hz, 1H, H‐2), 3.97 (dd, *J* = 12.2, 2.2 Hz, 1H, H‐6), 3.83 (dd, *J* = 12.2, 5.8 Hz, 1H, H‐6), 3.81–3.77 (m, 2H, H‐3, H‐4), 3.59 (qt, *J* = 6.3, 5.1 Hz, 1H, H‐5).

^13^C NMR (126 MHz, MeOD) δ 148.29 (dd, *J* = 246.5, 14.2 Hz), 147.42 (dd, *J* = 253.4, 15.0 Hz), 138.58 (t, *J* = 3.0 Hz), 128.16 (d, *J* = 4.0 Hz), 123.48 (d, *J* = 11.4 Hz), 122.40 (t, *J* = 3.7 Hz), 120.05 (d, *J* = 10.3 Hz), 108.63 (d, *J* = 17.7 Hz), 87.00, 80.13, 73.46, 70.81, 66.25, 61.10.

HRMS calculated for [C_14_H_15_BrF_2_N_3_O_5_]^+^ (M + H^+^): 442.0165, found 442.0163.

### 4.4 1‐[4‐(4‐Chloro‐3,5‐Difluorophenyl)‐1H‐(1,2,3)‐Triazol‐1‐Yl]‐1‐Deoxy‐β‐D‐Mannopyranose 7

Compound **9** (45 mg, 0.12 mmol) was added to a round‐bottom flask, dissolved in ACN (5 mL), and put under a N_2_ atmosphere. To this, CuI (2 mg, 0.01 mmol, 0.1 eq.), Et_3_N (80 µL, 0.6 mmol, 5 eq.), and ((4‐chloro‐3,5‐difluorophenyl)ethynyl)trimethylsilane (59 mg, 0.24 mmol, 2 eq.) were added and the solution was set to stir at rt for 18 h, after which TLC and LCMS indicated full conversion of the starting material. The solution was concentrated and the crude material dissolved in MeOH (9 mL) to which a solution of NaOMe in MeOH (1 M, 1 mL) was added. The mixture was set to stir at rt for 1 h, after which TLC indicated full conversion of the starting material. DOWEX 50W was added to neutralize the reaction mixture and was then filtered off. The filtrate was collected and concentrated under reduced pressure before purification using prep‐HPLC. Compound **7** (20 mg, 0.05 mmol, 44%) was afforded as a white amorphous solid after lyophilization.

^1^H NMR (400 MHz, CD_3_OD) δ 8.71 (s, 1H, triazole‐H), 7.70–7.59 (m, 2H, Ar‐H), 6.09 (d, *J* = 1.3 Hz, 1H, H‐1), 4.20 (t, *J* = 1.9 Hz, 1H), 3.98 (dd, *J* = 12.1, 2.3 Hz, 1H, H‐2), 3.88–3.74 (m, 4H, H‐3, H‐4, H‐6), 3.59 (tdd, *J* = 5.9, 4.0, 2.3 Hz, 1H, H‐5).

^13^C NMR (101 MHz, CD_3_OD) δ 159.17 (dd, *J* = 248.6, 3.8 Hz), 143.95, 131.54 (d, *J* = 9.8 Hz), 121.93, 109.18–108.25 (m), 87.05, 80.06, 73.47, 70.82, 66.32, 61.19.

HRMS calculated for [C_14_H_15_N_3_O_5_F_2_Cl]^+^ (M + H^+^): 378.0668, found 378.0669.

### 4.5 Methyl 1‐Azido‐β‐D‐Mannuronate 11

To a round bottom flask containing **10** [19] (1.64 g, 7.99 mmol), NaBr (82.25 mg, 0.80 mmol, 0.1 eq.), and TEMPO (25 mg, 0.16 mmol, 0.02 eq.) were added and then dissolved in a saturated aqueous solution of NaHCO_3_ (100 mL). The solution was cooled to 0°C before adding NaOCl (11 mL, 13%–15%). The mixture was slowly warmed to room temperature and set to stir overnight after which TLC indicated only partial conversion. More NaOCl (23 mL) was titrated in until TLC showed full conversion of the starting material. The mixture was neutralized using 1 M HCl and then concentrated under reduced pressure. To the solid residue, MeOH (50 mL) and DOWEX 50W (1.3 g) were added and the solution was set to stir at rt. The solution required further acidification and HCl was added (37% 1 mL) after which the reaction proceeded. After stirring overnight, TLC indicated full conversion of the starting material. The crude material was concentrated under reduced pressure and filtered through a short plug of silica using DCM:MeOH (5:1) to remove excess salts and then further purified using column chromatography (5:1) which afforded **11** as a transparent syrup (1.2 g, 5.15 mmol, 64%).

^1^H NMR (500 MHz, D_2_O) δ 4.99 (d, *J* = 1.2 Hz, 1H, H‐1), 4.08 (d, *J* = 9.9 Hz, 1H, H‐5), 4.05 (dd, *J* = 3.2, 1.2 Hz, 1H, H‐2), 3.89–3.81 (m, 4H, CH_3_, H‐4), 3.71 (dd, *J* = 9.7, 3.3 Hz, 1H, H‐3).

^13^C NMR (101 MHz, D_2_O) δ 170.48, 87.76, 76.47, 72.21, 70.42, 67.51, 53.04.

LRMS Calculated for [C_7_H_11_N_3_O_6_Na]^+^ (M + Na^+^): 256.05. Found 256.1.

### 4.6 Methyl 1‐Azido‐2,3‐O‐Isopropylidene‐β‐D‐Mannuronate 12

Compound **11** (345 mg, 1.48 mmol) was dissolved in 2,2‐dimethoxypropane (10 mL) and camphorsulfonic acid (19 mg, 0.08 mmol, 0.06 eq.) were added. The mixture was set to stir at rt overnight. The crude was concentrated and purified using gradient column chromatography (2:1–1:1 heptane:EtOAc) which afforded **12** as a white solid (332 mg, 1.22 mmol, 82%).

^1^H NMR (400 MHz, CDCl_3_) δ 5.08 (d, *J* = 2.9 Hz, 1H, H‐1), 4.34 (dd, *J* = 6.4, 2.9 Hz, 1H, H‐2), 4.32–4.20 (m, 2H, H‐3, H‐4), 4.05 (d, *J* = 7.8 Hz, 1H, H‐5), 3.85 (s, 3H, OMe), 2.91 (d, *J* = 3.1 Hz, 1H, OH), 1.55 (s, 3H, Me), 1.39 (s, 3H, Me). ^13^C NMR (101 MHz, CDCl_3_) δ 170.13, 111.31, 85.33, 75.31, 74.05, 69.37, 52.90, 26.81, 25.58.

### 4.7 Methyl 1‐[4‐(4‐Chloro‐3,5,‐Difluorophenyl)‐1H‐Triazolyl]‐2,3‐O‐Isopropylidene‐β‐D‐Mannuronate 13

Compound **12** (194 mg, 0.71 mmol) was added to a round bottom flask and dissolved in ACN (20 mL) put under a N_2_ atmosphere. To the solution Et_3_N (0.47 mL, 3.55 mmol, 5 eq.), CuI (13 mg, 0.07 mmol, 0.1 eq.), and (4‐chloro‐3,5‐difluorophenyl)‐trimethylsilylacetylene (261 mg, 1.06 mmol, 1.5 eq.). The reaction mixture was set to continue stir at room temperature for 5h, after which TLC and LCMS indicated full conversion of the starting material. The reaction mixture was concentrated and a solution of NH_4_Cl:NH_3_OH (50 mL, 9:1 ratio) was added and subsequently extracted using DCM (3x25 mL). The combined organic layers were dried using MgSO_4_ and concentrated in vacuo. The crude material was further purified using a Biotage silica cartridge and gradient elution (25%–75% EtOAc in heptane) which afforded **13** as a white solid (174 mg, 0.39 mmol, 55%).

^1^H NMR (400 MHz, CD_3_OD) δ 8.67 (s, 1H, triazole‐H), 7.71–7.62 (m, 2H, Ar‐H), 6.63 (d, *J* = 2.0 Hz, 1H, H‐1), 4.64–4.50 (m, 4H, H‐2, H‐3, H‐5, OH), 4.38 (t, *J* = 4.2 Hz, 1H, H‐4), 3.35 (s, 3H, OMe), 1.54 (s, 3H, Me), 1.32 (s, 3H, Me).

^13^C NMR (101 MHz, CD_3_OD) δ 171.15, 161.80, 159.37, 159.33, 145.65, 132.82, 123.47, 112.53, 110.37, 110.13, 110.11, 109.75, 84.30, 78.98, 76.97, 74.63, 68.44, 26.06, 24.87.

LRMS calculated for [C_18_H_19_ClF_2_N_3_O_6_]^+^ (M + H^+^): 446.09, found 446.0.

### 4.8 Methyl 1‐[4‐(4‐Chloro‐3,5‐Difluorophenyl)‐1H‐Triazolyl]‐2,3‐O‐Isopropylidene‐β‐D‐Mannuronate 14

Methyl ester **13** was added to a round bottom flask and dissolved in MeOH (8 mL) to which a solution of methanolic ammonia (7 M, 2 mL) was added. After ca 4h, the reaction was complete by TLC and the mixture was concentrated in vacuo. The crude **14** was used in the next reaction without further purification.

### 4.9 1‐[4‐(4‐Chloro‐3,5‐Difluorophenyl)‐1H‐Triazolyl]‐6‐Deoxy‐6‐[1‐(5‐Chloro‐2‐(Trifluoromethyl)phenyl)‐3‐Methyl‐1,2,4‐Triazolyl]‐β‐D‐Mannopyranoside 8

To a round bottom flask containing **14** (69 mg, 0.16 mmol), dioxane (5 mL), and DMA–DMA (100 µL, 0.62 mmol, 3.8 eq.) were added. The solution was set to stir at 80°C and after ca 6 h near full conversion was observed. The reaction mixture was concentrated under reduced pressure and then redissolved in AcOH (1 mL) to which (5‐chloro‐2‐(trifluoromethyl)phenyl)hydrazine (101 mg, 0.48 mmol, 3 eq.) was added. The solution was stirred at 80°C for 1 h, after which LCMS indicated full conversion of the intermediate **15**. To the solution, H_2_O (1 mL) was added, and the mixture was set to continue stir at 80°C for 64 h, after which LCMS indicated that the acetonide had been removed. The crude mixture was concentrated and purified using gradient flash chromatography (3:1–1:1 heptane:EtOAc) and then further purified using preparatory HPLC, which after lyophilization, afforded **8** as a white amorphous solid (7 mg, 0.01 mmol, 7%).

^1^H NMR (500 MHz, MeOD) δ 8.66 (s, 1H, Ar‐H), 7.89 (d, *J* = 8.5 Hz, 1H, Ar‐H), 7.81 (s, 1H, Ar‐H), 7.69 (s, 1H, Ar‐H), 7.68–7.61 (m, 2H, Ar‐H), 6.15 (d, *J* = 1.3 Hz, 1H, H‐1), 4.53 (d, *J* = 9.6 Hz, 1H, H‐5), 4.28 (s, 1H, H‐4), 4.12 (dd, *J* = 3.2, 1.3 Hz, 1H, H‐2), 3.80 (dd, *J* = 9.5, 3.1 Hz, 1H, H‐3), 2.47 (s, 3H, Me).

HRMS calculated for [C_23_H_18_Cl_2_F_5_N_6_O_3_]^+^ (M + H^+^): 607.1245, found 607.0687.

### 4.10 Methyl (4R, 7aS)‐4‐Azido‐2,2‐Dimethyl‐3a, 7a‐Dihydro‐4H‐[1,3]dioxolo[4,5‐c]pyran‐6‐Carboxylate 16

Compound **12** (25 mg, 0.09 mmol) was added to a round bottom flask and dissolved in pyridine (1 mL). To this, MsCl (21 µL, 0.27 mmol, 3 eq.) was added and the solution was set to stir at rt overnight (18 h). As TLC indicated only partial conversion, more MsCl (42 µL 0.54 mmol, 6 eq.) was added which resulted in full conversion of the starting material according to TLC. The crude mixture was diluted with EtOAc (10 mL) and washed with sat. NaHCO_3_ (2 × 10 mL). The organic layer was collected and dried using MgSO_4_ and then concentrated under reduced pressure. The crude mixture was then filtered through a short plug of silica (1:1 heptane:EtOAc) which afforded the intermediate as an oily residue (22 mg, 0.06 mmol, 68%).

To a round bottom flask containing the mesylate intermediate, basic Al_2_O_3_ (220 mg) was added, and the mixture was dispersed in 2,6‐lutidine (2 mL). The mixture was then heated to 40°C and stirred overnight, after which TLC indicated full conversion of the starting material. The crude was then concentrated and filtered through a short plug of silica (2:1 heptane:EtOAc) which afforded **16** in quantitative yields.

^1^H NMR (400 MHz, CDCl_3_) δ 6.29 (d, *J* = 3.7 Hz, 1H, H‐1), 5.28 (d, *J* = 2.7 Hz, 1H, H‐4), 4.75 (dd, *J* = 6.4, 3.7 Hz, 1H, H‐2), 4.40 (dd, *J* = 6.4, 2.7 Hz, 1H, H‐3), 3.86 (s, 3H, COOMe), 1.53 (s, 3H, Me), 1.43 (s, 3H, Me).

^13^C NMR (101 MHz, CDCl_3_) δ 161.86, 142.46, 111.39, 110.55, 85.49, 72.77, 68.33, 52.69, 26.90, 26.02.

### 4.11 Methyl (4R, 7aS)‐4‐(4‐(4‐Chloro‐3,5‐Difluorophenyl)‐1H‐1,2,3‐Triazol‐1‐Yl)‐2,2‐Dimethyl‐3a, 7a‐Dihydro‐4H‐[1,3]dioxolo[4,5‐c]pyran‐6‐Carboxylate 17

Compound **16** (16 mg, 0.06 mmol was added to a round bottom flask, dissolved in ACN (5 mL) and put under a N_2_ atmosphere. To the solution, CuI (2 mg, 0.01 mmol, 0.2 eq.), Et_3_N (0.03 mL, 0.19 mmol, 3 eq.), and ((4‐chloro‐3,5‐difluorophenyl)ethynyl)trimethylsilane (28 mg, 0.12 mmol, 2 eq.) were added and the solution was set to stir at rt for 72 h, after which TLC and LCMS indicated full conversion of the starting material. The crude mixture was concentrated and then diluted with a mixture of sat. NH_4_Cl and NH_4_OH (9:1, 10 mL) and then extracted with DCM (3 × 10 mL). The organic layers were collected and dried using MgSO_4_ before removing the solvents under reduced pressure. The crude was purified using column chromatography (2:1 Heptane:EtOAc) which afforded **17** as a white solid (20 mg, 0.05 mmol, 75%).

^1^H NMR (400 MHz, CDCl_3_) δ 8.36 (s, 1H, triazole‐H), 7.60–7.45 (m, 2H, Ar‐H), 6.46 (d, *J* = 1.3 Hz, 1H, H‐1), 6.25 (dd, *J* = 3.3, 1.2 Hz, 1H, H‐4), 5.11 (dd, *J* = 5.8, 3.3 Hz, 1H, H‐3), 4.61 (dt, *J* = 5.8, 1.4 Hz, 1H, H‐2), 3.85 (s, 3H, COOMe), 1.54 (s, 3H, Me), 1.42 (s, 3H, Me).

^13^C NMR (101 MHz, CDCl_3_) δ 161.31, 159.29 (dd, *J* = 250.0, 4.0 Hz), 145.66, 142.64, 130.54 (d, *J* = 9.6 Hz), 120.86, 115.22, 112.84, 111.24, 109.82–108.98 (m), 84.37, 72.82, 70.10, 52.82, 28.06, 26.82.

LRMS calculated for [C_18_H_17_ClF_2_N_3_O_5_]^+^ (M + H^+^): 428.08, found 428.1.

### 4.12 Methyl 1‐[4‐(4‐Chloro‐3,5‐Difluorophenyl)‐1H‐1,2,3‐Triazol‐1‐Yl]‐4‐Deoxy‐2,3‐O‐Isopropylidene‐β‐D‐Mannuronate 18

Compound **17** (13 mg, 0.03 mmol) was added to a round bottom flask and dissolved in 5 mL EtOH. To the solution, Pd/C (10%, 4 mg, 0.004 mmol, 0.1 eq.) was added and then the flask was put under a H_2_ atmosphere. The mixture was set to stir at rt overnight (16 h), after which LCMS indicated full conversion of the starting material. The crude was filtered through a pad of celite. The filtrate was collected, concentrated and then further purified by filtration through a short plug of SiO_2_ (3:2 heptane:EtOAc) which afforded **18** as a white solid (13 mg, quant.).

^1^H NMR (400 MHz, CDCl_3_) δ 8.31 (s, 1H, triazole‐H), 7.59–7.45 (m, 2H, Ar‐H), 6.19 (d, *J* = 2.0 Hz, 1H, H‐1), 4.66 (td, *J* = 6.2, 4.5 Hz, 1H, H‐3), 4.54 (t, *J* = 5.9 Hz, 1H, H‐5), 4.43 (dd, *J* = 6.6, 2.1 Hz, 1H, H‐2), 2.49–2.27 (m, 2H, H‐4), 1.59 (s, 3H, Me), 1.32 (s, 3H, Me).

^13^C NMR (101 MHz, CDCl_3_) δ 170.32, 161.49–157.22 (m), 145.23, 131.00, 121.34, 109.43 (dd, *J* = 24.0, 2.4 Hz), 83.86, 72.78, 71.92, 71.29, 52.67, 26.48, 25.29.

LRMS calculated for [C_18_H_19_ClF_2_N_3_O_5_]^+^ (M + H^+^): 430.10, found 430.0.

### 4.13 1‐[4‐(4‐Chloro‐2,3‐Difluorophenyl)‐1H‐1,2,3‐Triazol‐1‐Yl]‐4‐Deoxy‐2,3‐O‐Isopropylidene‐β‐D‐Mannuronamide 19

Methyl ester **18** (13 mg, 0.03 mmol) was added to a round bottom flask and dissolved in MeOH (4 mL) to which a solution of methanolic ammonia (7 M, 1 mL) was added. After stirring for 18 h, the reaction was completed by TLC and the crude was concentrated in vacuo. The crude **19** was used in the next reaction without further purification.

### 4.14 (2R, 3S,4S, 6S)‐6‐(1‐(5‐Chloro‐2‐(Trifluoromethyl)phenyl)‐3‐Methyl‐1H‐1,2,4‐Triazol‐5‐Yl)‐2‐(4‐(4‐Chloro‐3,5‐Difluorophenyl)‐1H‐1,2,3‐Triazol‐1‐Yl)tetrahydro‐2H‐Pyran‐3,4‐Diol 21

To a round bottom flask containing crude **19** (28 mg, 0.07 mmol), 1,4‐dioxane (5 mL), and DMA–DMA (100 µL, 0.62 mmol, 9 eq.) were added. The solution was set to stir at 80°C for 2h, after which only partial conversion was observed. The temperature was increased to reflux and more DMA–DMA was added (100 µL) and the solution was set to stir for 48 h, after which near full conversion was observed on TLC. The crude was concentrated and then redissolved in AcOH (1 mL) to which (5‐chloro‐2‐(trifluoromethyl)phenyl)hydrazine (43 mg, 0.20 mmol, 3 eq.) was added. The solution was stirred at 80°C for 1 h after which LCMS indicated full conversion of the intermediate to **20**. To the solution containing **20**, H_2_O (1 mL) was added, and the mixture was set to continue stir at 80°C for 64 h, after which LCMS indicated that the acetonide had been removed. The crude mixture was concentrated and purified using gradient flash chromatography (3:1–1:1 heptane:EtOAc) and then further purified using preparatory HPLC, which after lyophilization, afforded **21** as a white amorphous solid (3 mg, 0.005 mmol, 8%).

^1^H NMR (400 MHz, CD_3_OD) δ 8.41 (s, 1H, triazole‐H), 7.73 (d, *J* = 8.4 Hz, 1H), 7.70–7.57 (m, 4H, Ar‐H), 5.93 (s, 1H, H‐1), 4.99 (d, *J* = 11.4 Hz, 1H), 4.08 (d, *J* = 11.0 Hz, 1H), 3.97 (s, 1H), 2.43 (s, 3H, Me), 2.38 (d, *J* = 12.6 Hz, 1H, H‐4), 2.23 (d, *J* = 13.3 Hz, 1H, H‐4).

HRMS calculated for [C_23_H_18_Cl_2_F_5_N_6_O_3_]^+^ (M + H^+^): 581.0754, found 591.0738.

### 4.15 (2R, 3S,4S)‐6‐(1‐(5‐Chloro‐2‐(Trifluoromethyl)phenyl)‐3‐Methyl‐1H‐1,2,4‐Triazol‐5‐Yl)‐2‐(4‐(4‐Chloro‐3,5‐Difluorophenyl)‐1H‐1,2,3‐Triazol‐1‐Yl)‐3,4‐Dihydro‐2H‐Pyran‐3,4‐Diol 23

To a round‐bottom flask containing **14** (78 mg, 0.18 mmol), 1,4‐dioxane (5 mL), and DMA–DMA (200 µL, 1.23 mmol, 7 eq.) were added. The solution was set to stir at reflux for 18 h, after which TLC indicated full conversion of the starting material. The mixture was concentrated under reduced pressure, and to the crude, 5‐chloro‐2‐(trifluoromethyl)phenyl hydrazine (76 mg, 0.36 mmol, 2 eq.) and AcOH (4 mL) were added. The solution was stirred for 1h at 80 C, after which full conversion of intermediate into **22** was observed by LCMS. To the solution containing **22**, H_2_O (2 mL) was added and then the solution was stirred at 80°C for 72hr after which the crude mixture was concentrated and purified using column chromatography (2:1–1:5 Heptane:EtOAc). The product was further purified using prep‐HPLC, which afforded **23** as an amorphous white solid (2 mg, 0.003 mmol, 2%).

^1^H NMR (500 MHz, CD_3_OD) δ 8.36 (s, 1H, triazole‐H), 7.66 (d, *J* = 8.0 Hz, 2H, Ar‐H), 7.62–7.54 (m, 2H, Ar‐H), 7.35 (d, *J* = 8.3 Hz, 1H, Ar‐H), 6.35 (s, 1H, H‐1), 6.04 (s, 1H, H‐4), 4.81 (dd, *J* = 4.5, 2.4 Hz, 1H, H‐3), 4.11 (dt, *J* = 4.2, 1.4 Hz, 1H, H‐2), 2.42 (s, 3H, Me).

^13^C NMR (126 MHz, CD_3_OD) δ 160.37, 158.15, 138.77 (d, J = 235.6 Hz), 129.97, 129.62, 127.56, 121.09, 108.92, 108.72, 108.31, 85.48, 65.40, 64.24, 11.88.

HRMS calculated for [C_23_H_16_Cl_2_F_5_N_6_O_3_]^+^ (M + H^+^): 589.0588, found 589.0581.

### 4.16 Molecular Dynamic Simulations

Complexes of **4**, **7**‐**8**, **21**, and **23** with galectin‐3 and galectin‐9N were built in Schrödinger Maestro (Release 2025–1, Schrödinger LLC). For galectin‐3, the X‐ray structure (pdb id 7XFA) of **4** bound to galectin‐3 was imported and prepared with the protein preparation workflow implemented in Schrödinger's Maestro. The mannosyls **7**‐**8**, **21**, and **23** were in turn built from the complex of **4**. For galectin‐9N, the X‐ray structure (pdb id 3WLU) of a seleno‐lactose derivative bound to galectin‐9N was imported and prepared with the protein preparation workflow implemented in Schrödinger's Maestro. Then, the pyranose ring of galactosyl **4** with the conformation as in the X‐ray with galectin‐3 (pdb id 7FXA) was overlayed with the galactopyranose ring of the seleno‐lactose derivative bound to galectin‐9N to generate a complex of **4** and galectin‐9N. The mannosyls **7**‐**8**, **21**, and **23** were in turn built from the complex of **4**.

MD simulations were performed using the Desmond software using the OPLS3 force field implemented in Schrödinger Maestro with default settings except for the duration, which was 200 ns. The O‐4 atom of **4**, the O‐2 atoms of **7**‐**8**, **21**, and **23,** and all stranded backbone atoms were subjected to light position restraint with a force constant of 1 kcal/mol/Å^2^. Trajectory cluster analyses were performed for all MD analyses in Desmond to identify preferred binding poses and ligand‐protein interactions.

## Supporting Information

Additional supporting information can be found online in the Supporting Information section.

## Author Contributions

**F.** **S.** designed structures, performed syntheses, and did fluorescence polarization experiments. **U.** **J.** **N.** conceptualized, recruited funding for, and supervised the project. Both authors interpreted data and wrote the manuscript.

## Funding

This work was supported by the Vetenskapsrådet (2025−04985); Kungliga Fysiografiska Sällskapet i Lund; Galecto Biotech AB.

## Conflicts of Interest

The authors declare no conflicts of interest.

## Supporting information

Supplementary Material

## Data Availability

The data that support the findings of this study are available from the corresponding author upon reasonable request.
