# Supplementary material for: β‐Mannosyl Triazoles as Mimics of Galactosyl Galectin‐3 and Galectin‐9 N‐Terminal Domain Inhibitors
Source: Chembiochem. 2026 Apr 13;27(7):e70319. doi: 10.1002/cbic.70319 (PMC13072086; doi:10.1002/cbic.70319)

β-Mannosyl triazoles as mimics of galactosyl galectin-3 and galectin-9 N-terminal domain inhibitors

Fredrik Sjövall and Ulf J. Nilsson*

Department of Chemistry, Lund University, Lund, Sweden

Figure S1 Trajectory clustering poses of MD of **7** complexes S2
Supplementary copies of nmr spectra S3

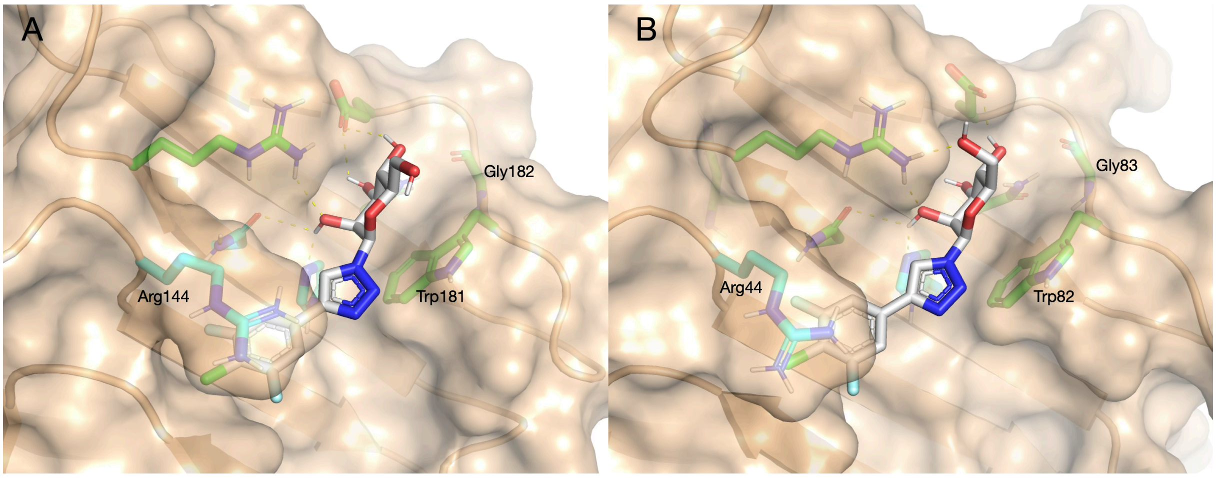

Figure S1. Poses from trajectory clustering of MD simulations of **7** in complex with A) galectin-3 and B) galectin-9N.

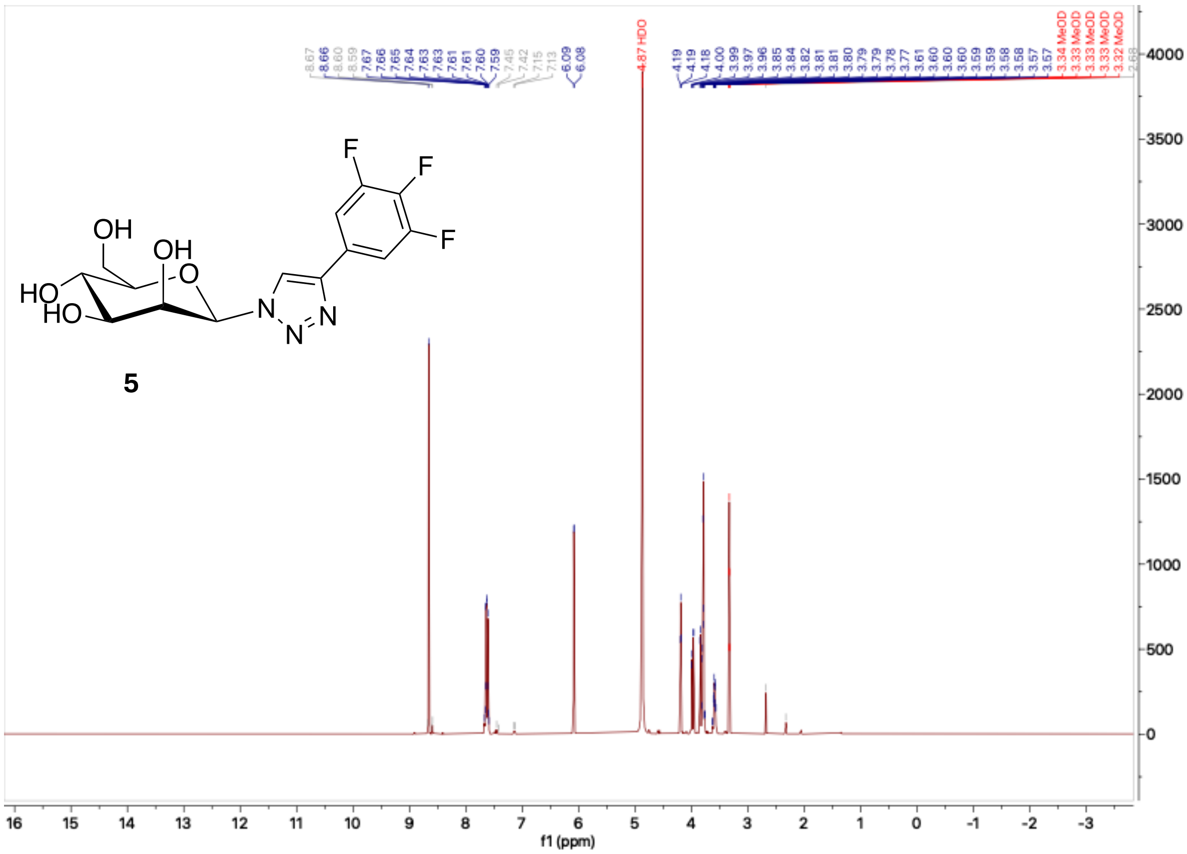
8.67 8.66 8.60 8.59 7.67 7.66 7.65 7.64 7.63 7.63 7.61 7.61 7.60 7.59 7.45 7.42 7.15 7.13 6.09 6.08
4.87 HDO
4.19 4.19 4.18 4.00 3.99 3.97 3.96 3.85 3.84 3.82 3.81 3.81 3.80 3.79 3.79 3.78 3.77 3.61 3.60 3.60 3.60 3.59 3.59 3.58 3.58 3.57 3.57
3.34 MeOD 3.33 MeOD 3.33 MeOD 3.33 MeOD 3.32 MeOD 2.68
OH
OH
O
HO
HO
N
N
N
N
F
F
F
5
f1 (ppm)

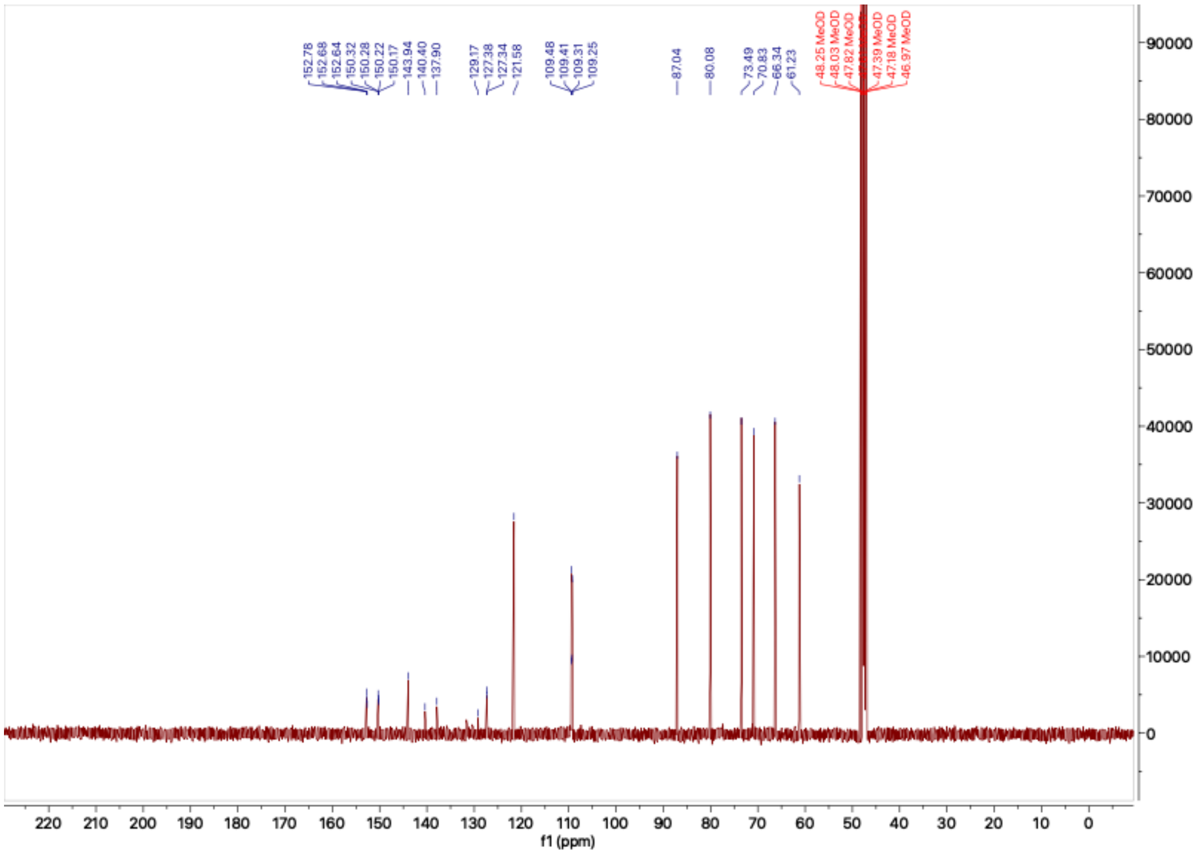
152.78 152.68 152.64 150.32 150.28 150.22 150.17 143.94 140.40 137.90 129.17 127.38 127.34 121.58 109.48 109.41 109.31 109.25 87.04 80.08 73.49 70.83 66.34 61.23
48.25 MeOD 48.03 MeOD 47.82 MeOD 47.39 MeOD 47.18 MeOD 46.97 MeOD
f1 (ppm)

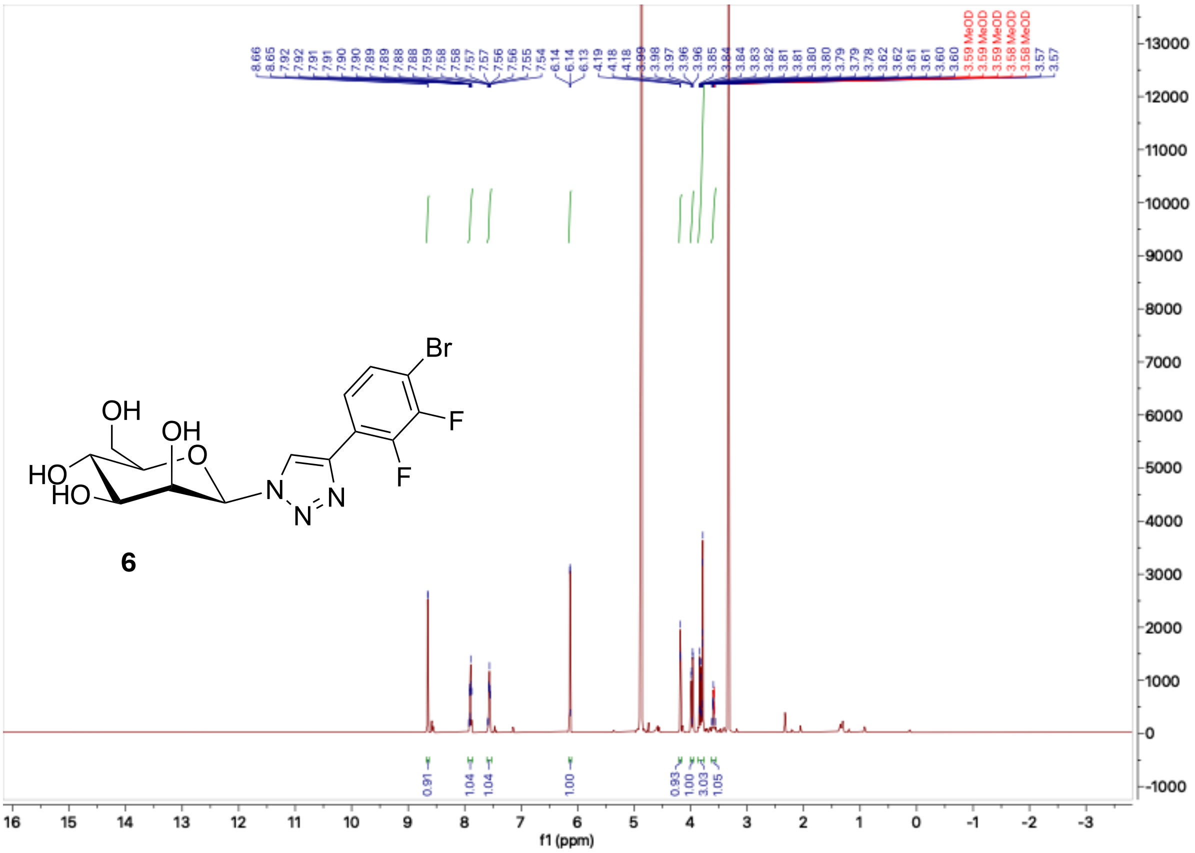

6

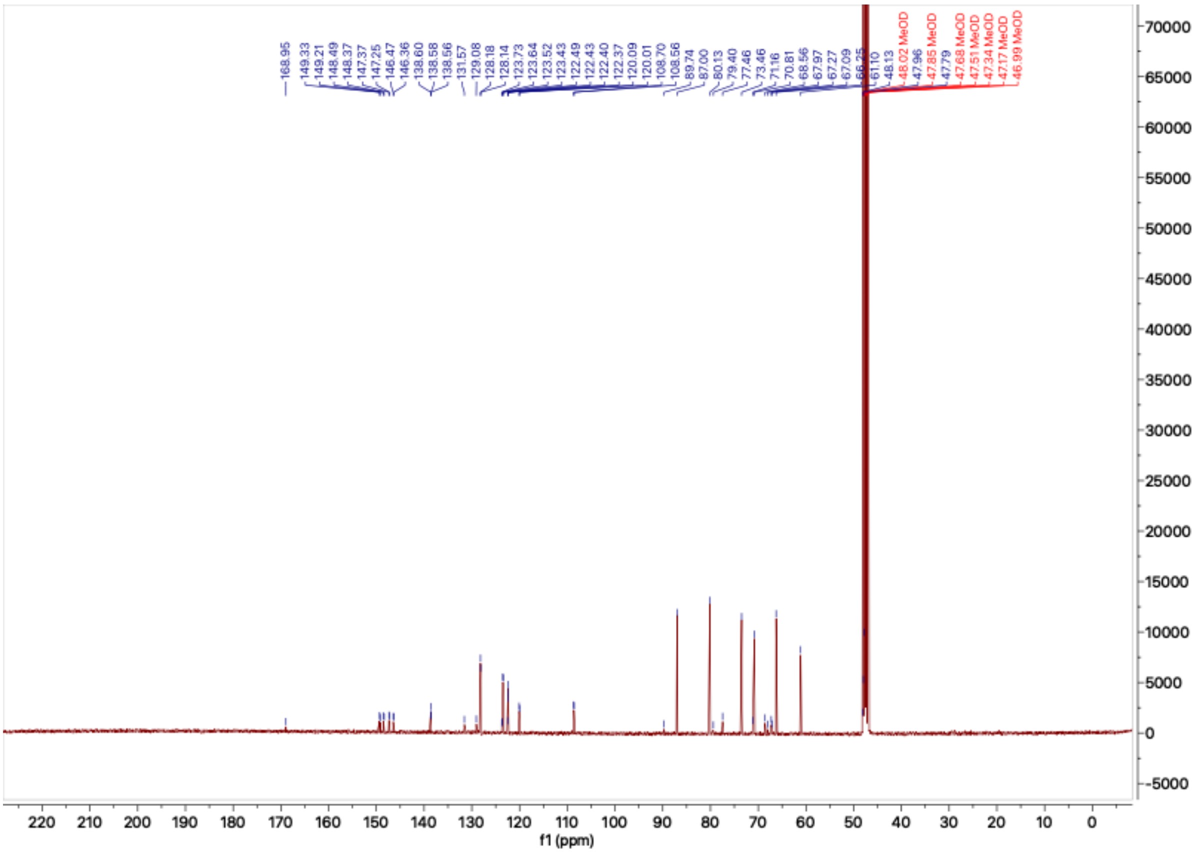

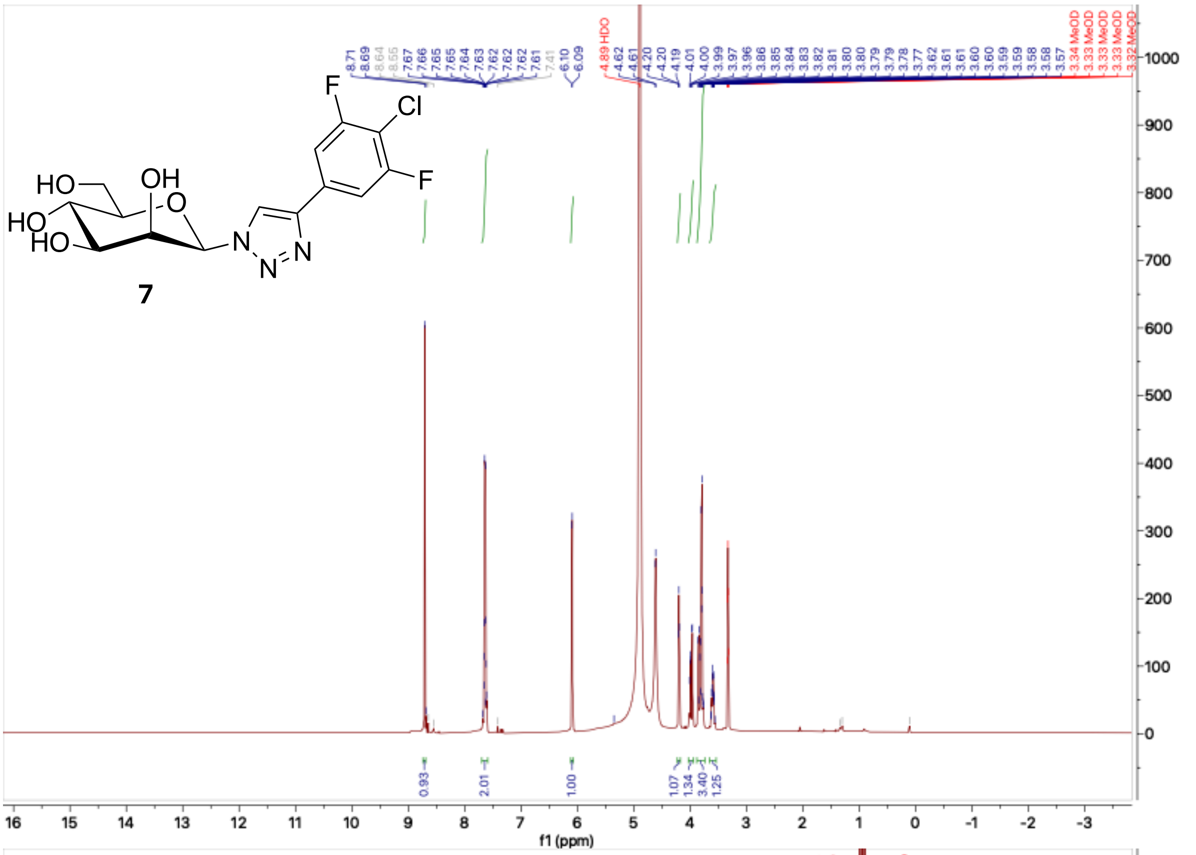
7
f1 (ppm)

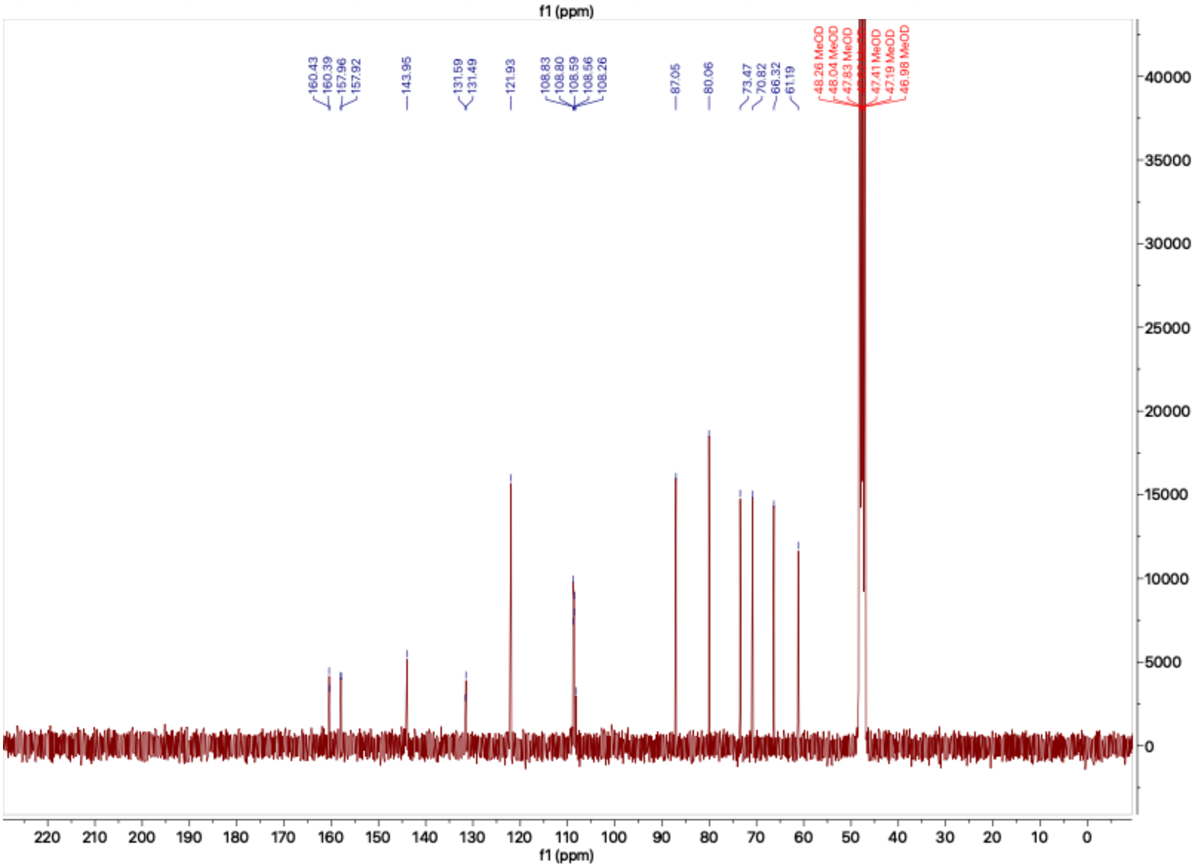
f1 (ppm)

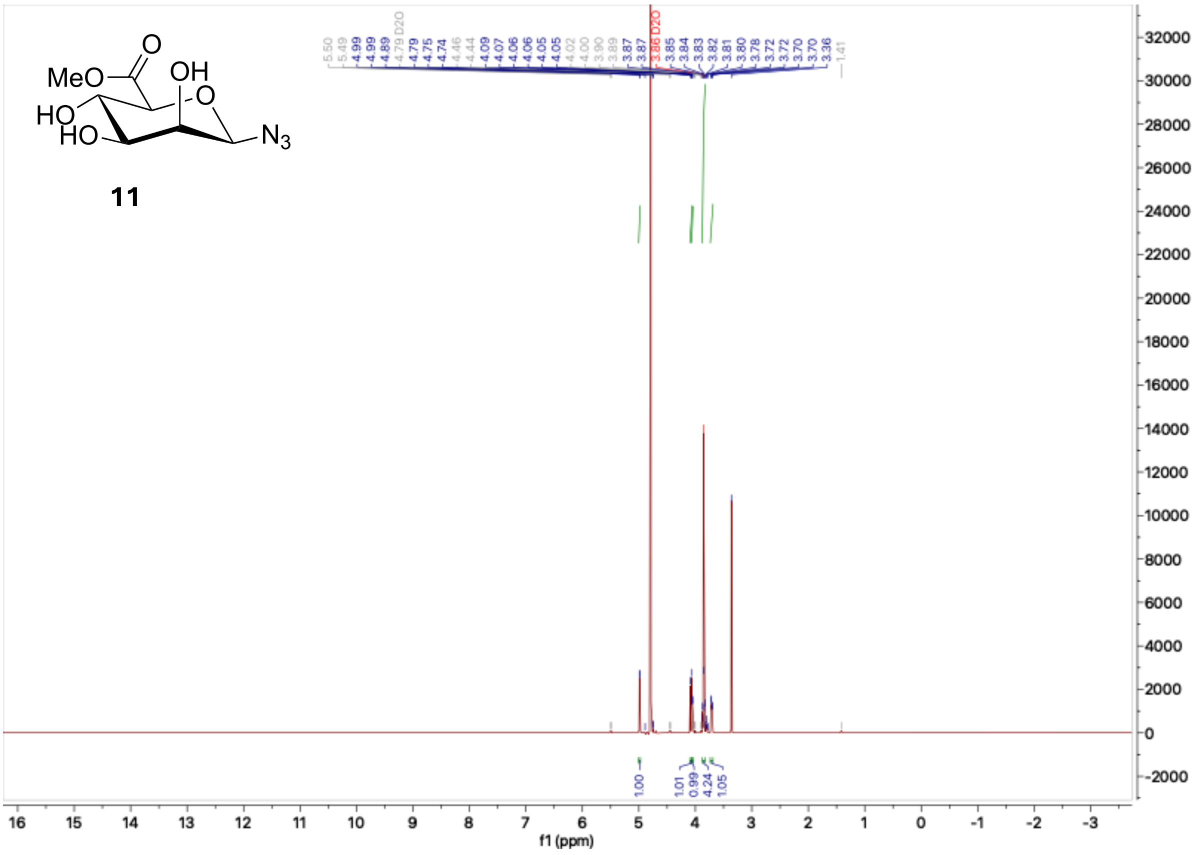
MeO
O
OH
O
HO
HO
N3
11
5.50 5.49 4.99 4.99 4.89 4.79 D2O 4.79 4.75 4.74 4.46 4.44 4.09 4.07 4.06 4.06 4.05 4.05 4.02 4.00 3.90 3.89 3.87 3.87 3.86 D2O 3.85 3.84 3.83 3.82 3.81 3.80 3.78 3.72 3.72 3.70 3.70 3.36 1.41
1.00
1.01
0.99
4.24
1.05
f1 (ppm)

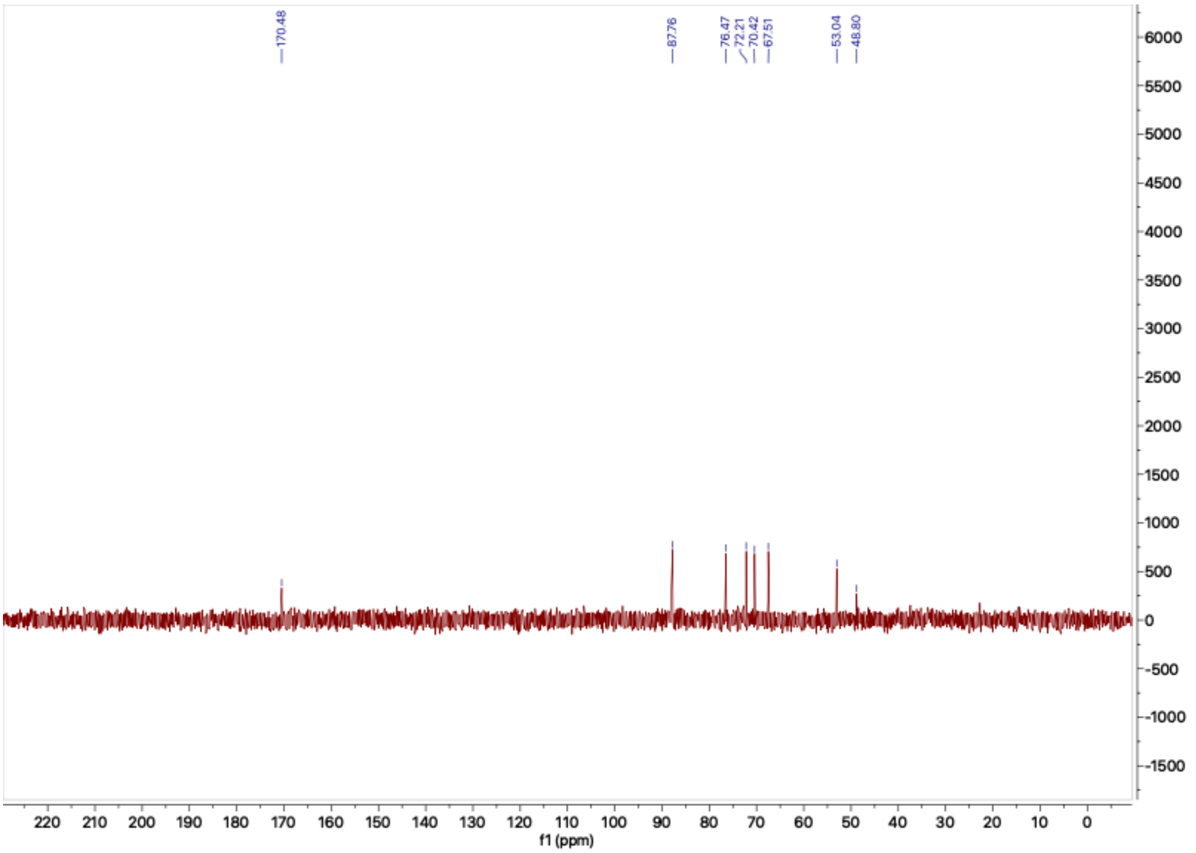
170.48
87.76
76.47
72.21
70.42
67.51
53.04
48.80
f1 (ppm)

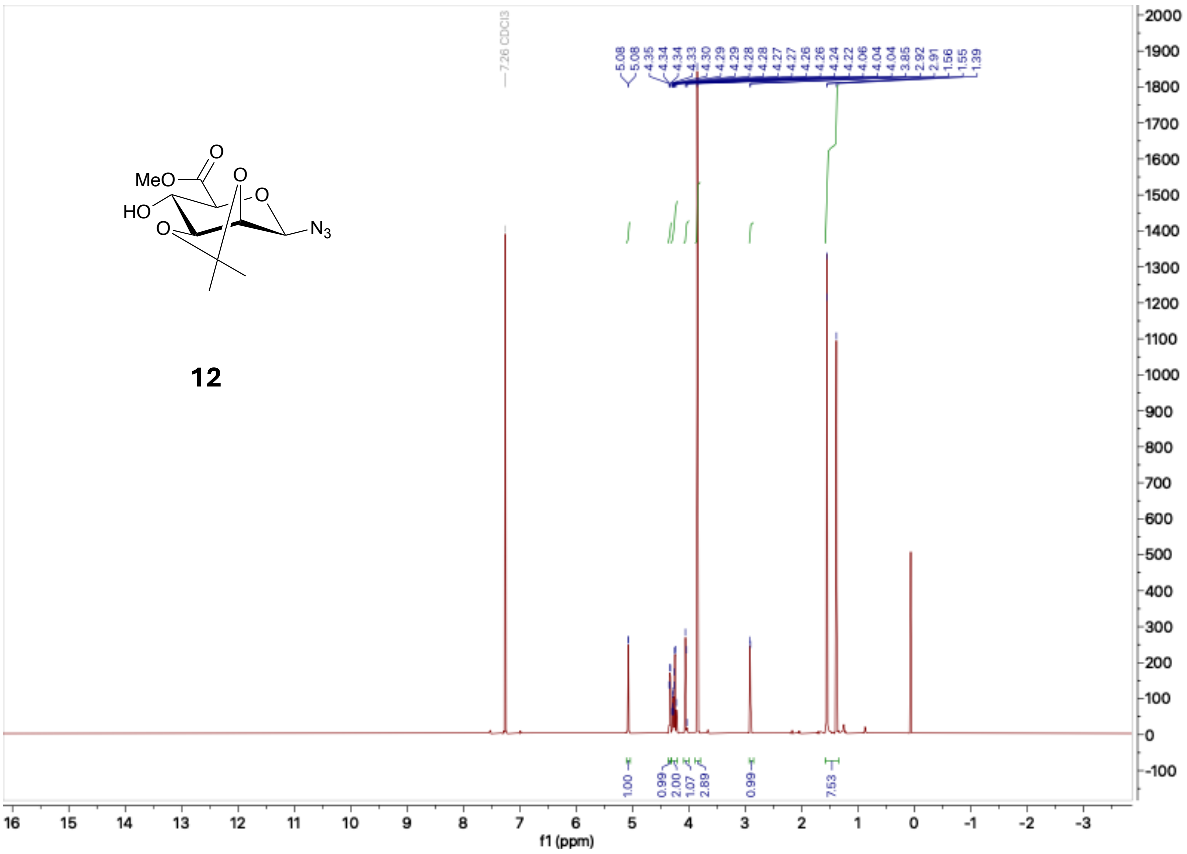

MeO
O
O
O
HO
O
N_3
7.26 CDCl3
5.08
5.08
4.35
4.34
4.34
4.33
4.30
4.29
4.29
4.28
4.28
4.27
4.27
4.26
4.26
4.24
4.22
4.06
4.04
4.04
3.85
2.92
2.91
1.56
1.55
1.39
1.00
0.99
2.00
1.07
2.89
0.99
7.53
f1 (ppm)

12

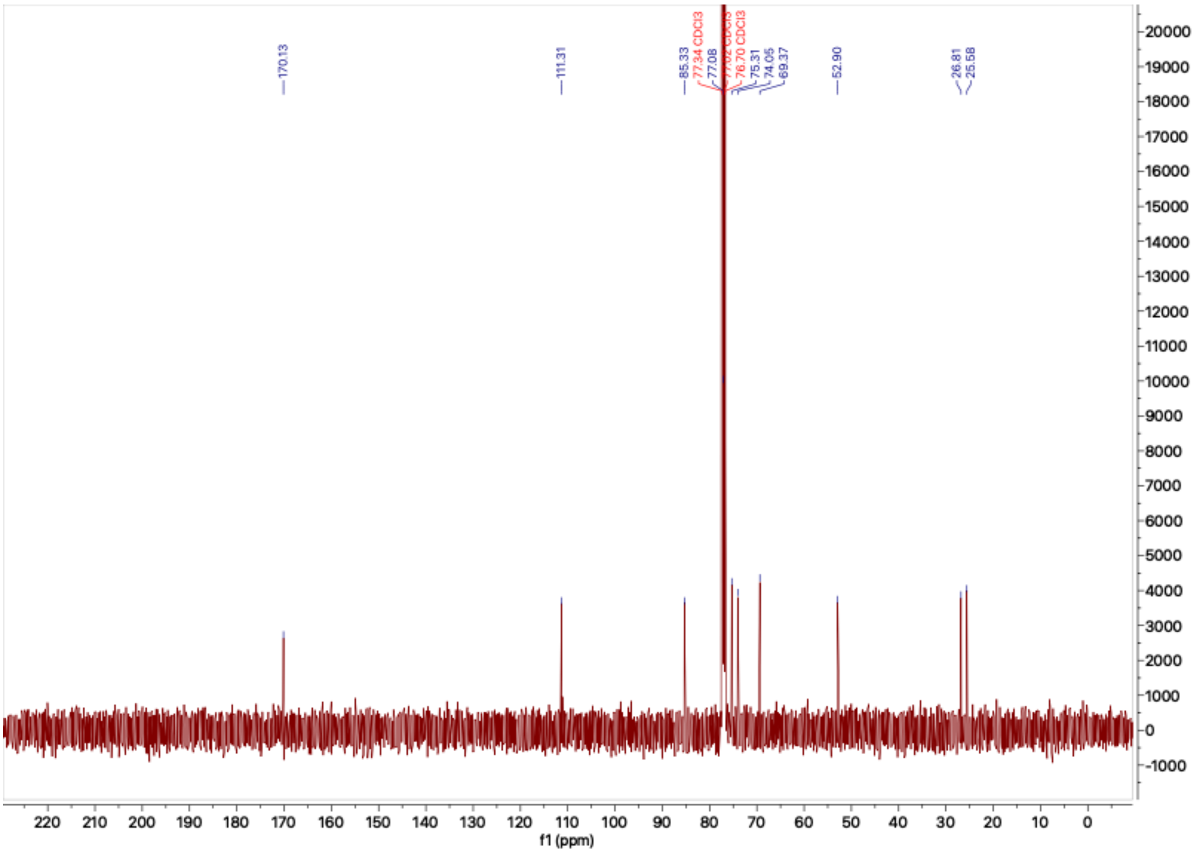

170.13
111.31
85.33
77.34 CDCl3
77.08
77.02 CDCl3
76.70 CDCl3
75.31
74.05
69.37
52.90
26.81
25.58
f1 (ppm)

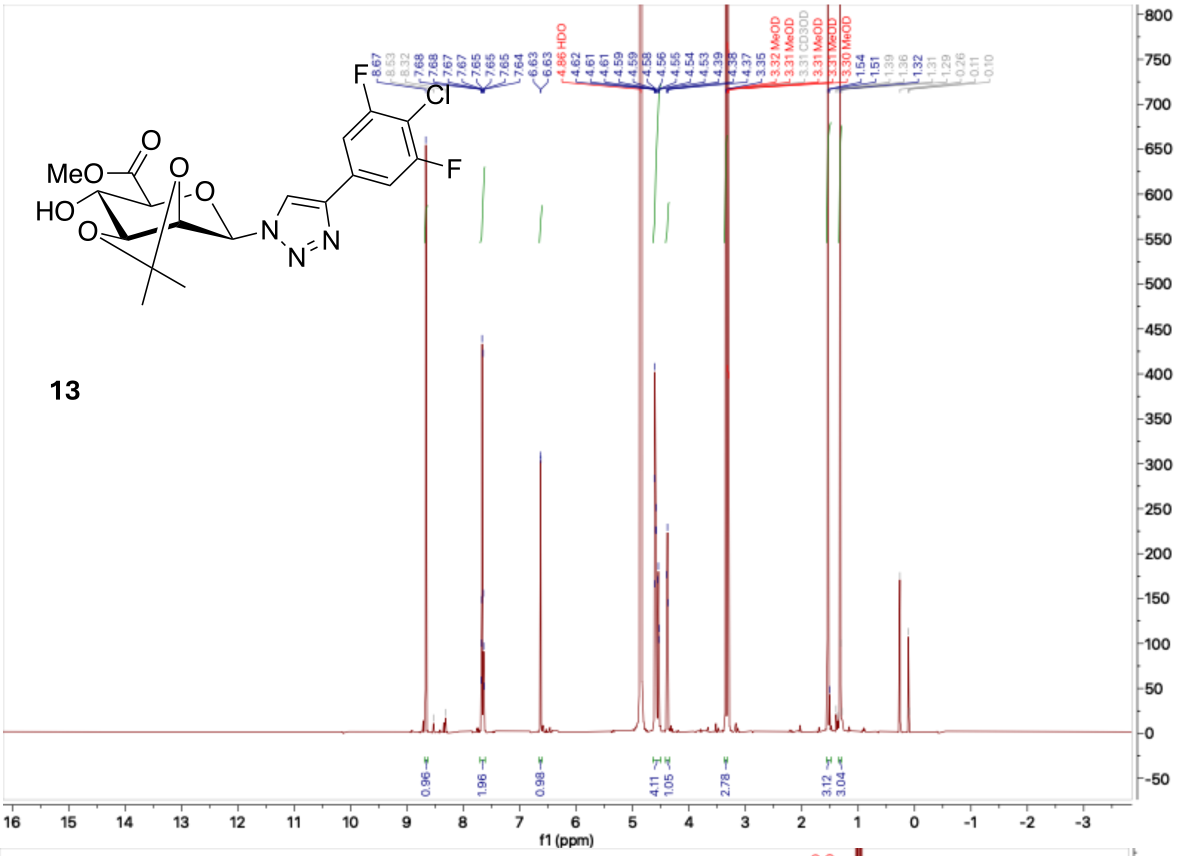

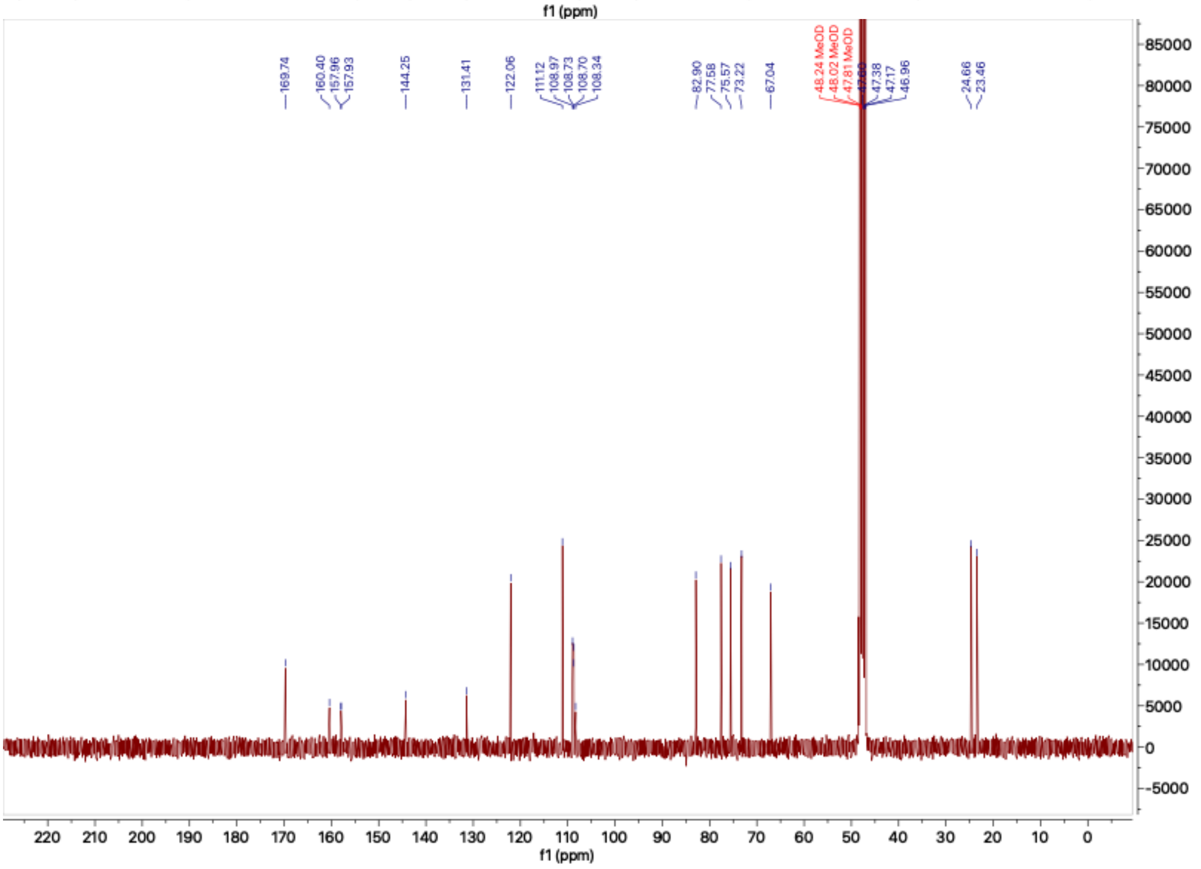

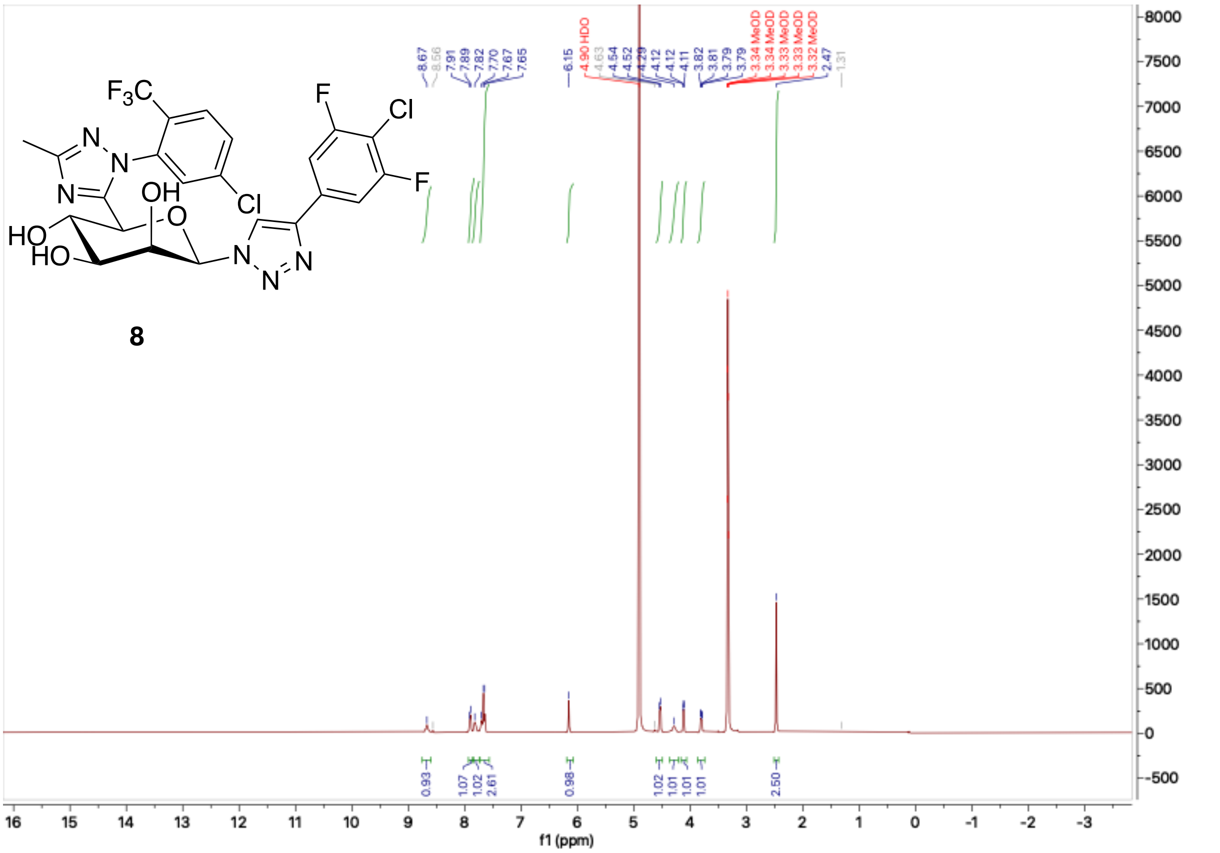

8
8.67
8.56
7.91
7.89
7.82
7.70
7.67
7.65
6.15
4.90 HDO
4.63
4.54
4.52
4.29
4.12
4.12
4.11
3.82
3.81
3.79
3.79
3.34 MeOD
3.34 MeOD
3.33 MeOD
3.33 MeOD
3.32 MeOD
2.47
1.31
0.93
1.07
1.02
2.61
0.98
1.02
1.01
1.01
1.01
2.50
f1 (ppm)

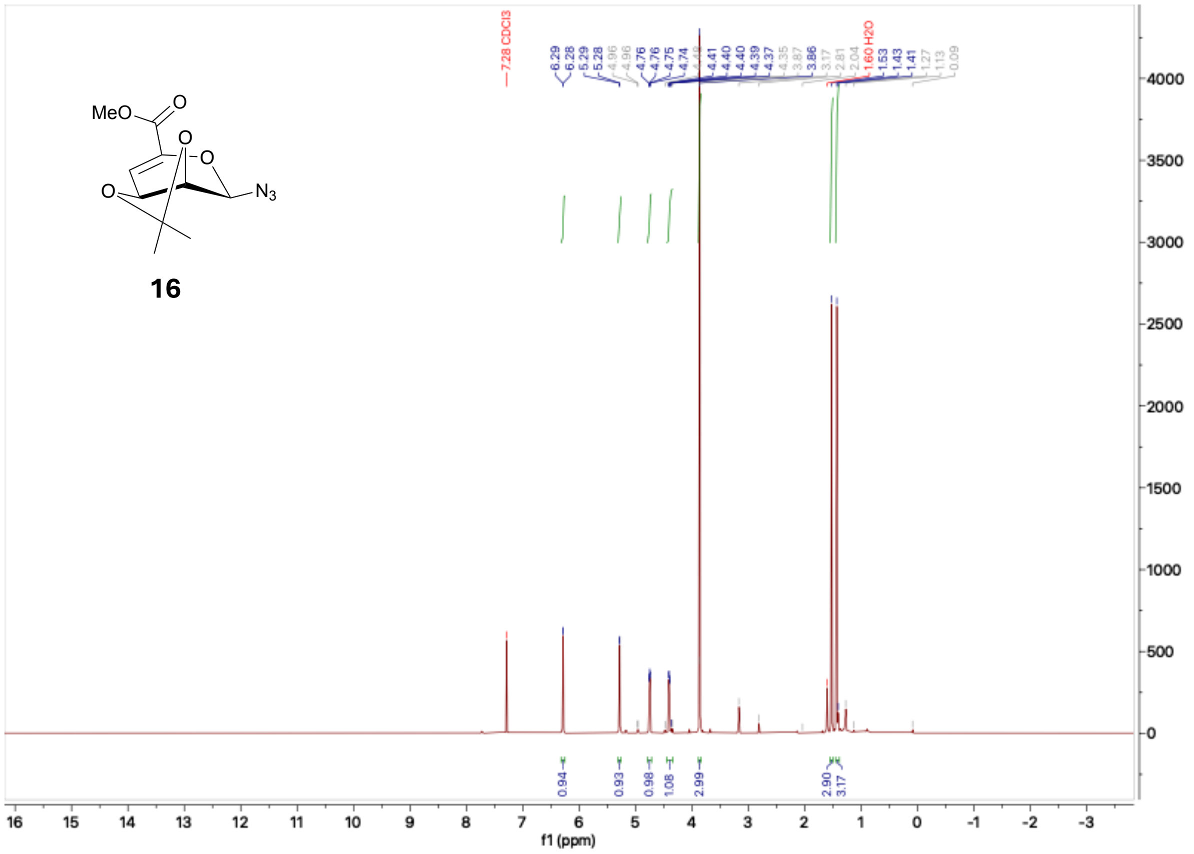
MeO
O
O
O
O
N3
7.28 CDCl3
6.29
6.28
5.29
5.28
4.96
4.96
4.76
4.76
4.75
4.74
4.41
4.40
4.40
4.39
4.37
4.35
3.87
3.86
3.17
2.81
2.04
1.60 H2O
1.53
1.43
1.41
1.27
1.13
0.09
0.94
0.93
0.98
1.08
2.99
2.90
3.17
f1 (ppm)

16

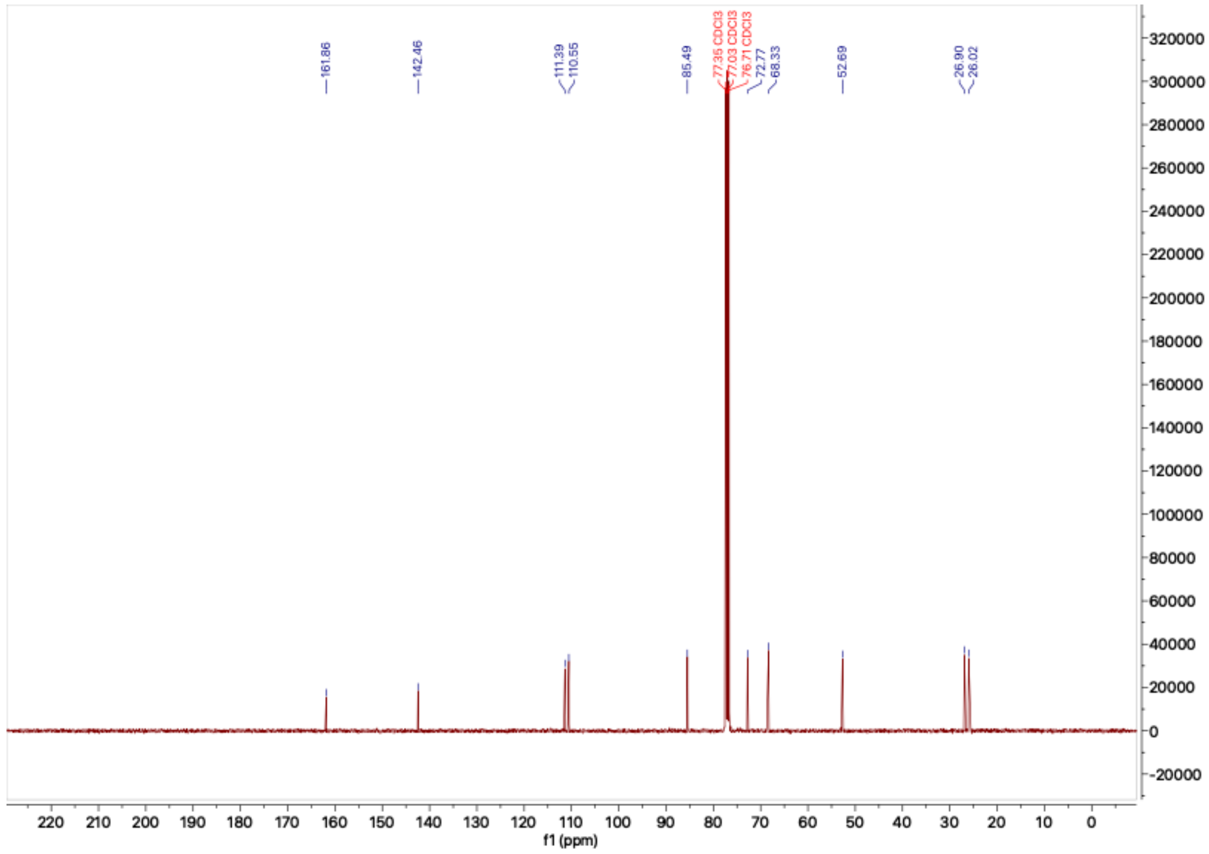
161.86
142.46
111.39
110.55
85.49
77.35 CDCl3
77.03 CDCl3
76.71 CDCl3
72.77
68.33
52.69
26.90
26.02
f1 (ppm)

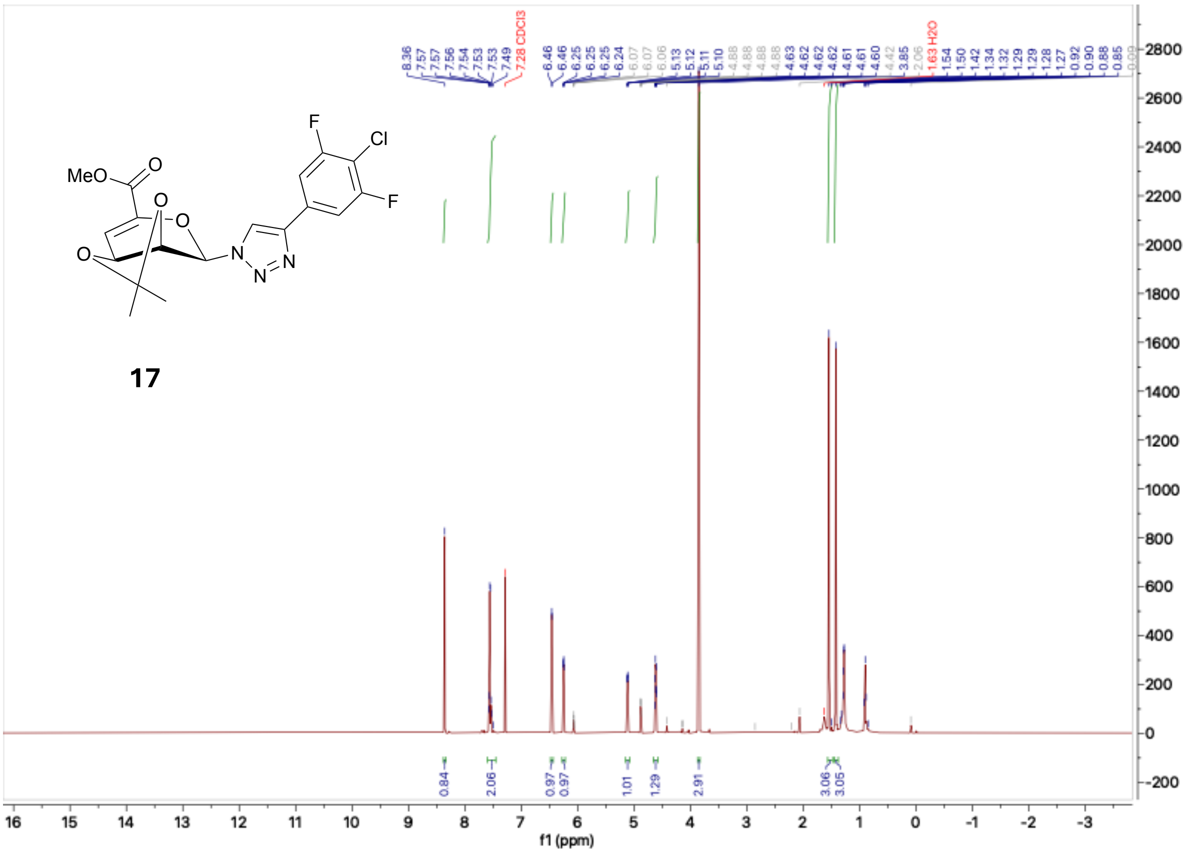

MeO
O
O
O
O
N
N
N
F
Cl
F
17
8.36
7.57
7.57
7.56
7.54
7.53
7.53
7.49
7.28 CDCl3
6.46
6.46
6.25
6.25
6.25
6.24
6.07
6.07
6.06
5.13
5.12
5.11
5.10
4.88
4.88
4.88
4.88
4.63
4.62
4.62
4.62
4.61
4.61
4.60
4.42
3.85
2.06
1.63 H2O
1.54
1.50
1.42
1.34
1.32
1.29
1.29
1.28
1.27
0.92
0.90
0.88
0.85
0.09
0.84
2.06
0.97
0.97
1.01
1.29
2.91
3.06
3.05
f1 (ppm)

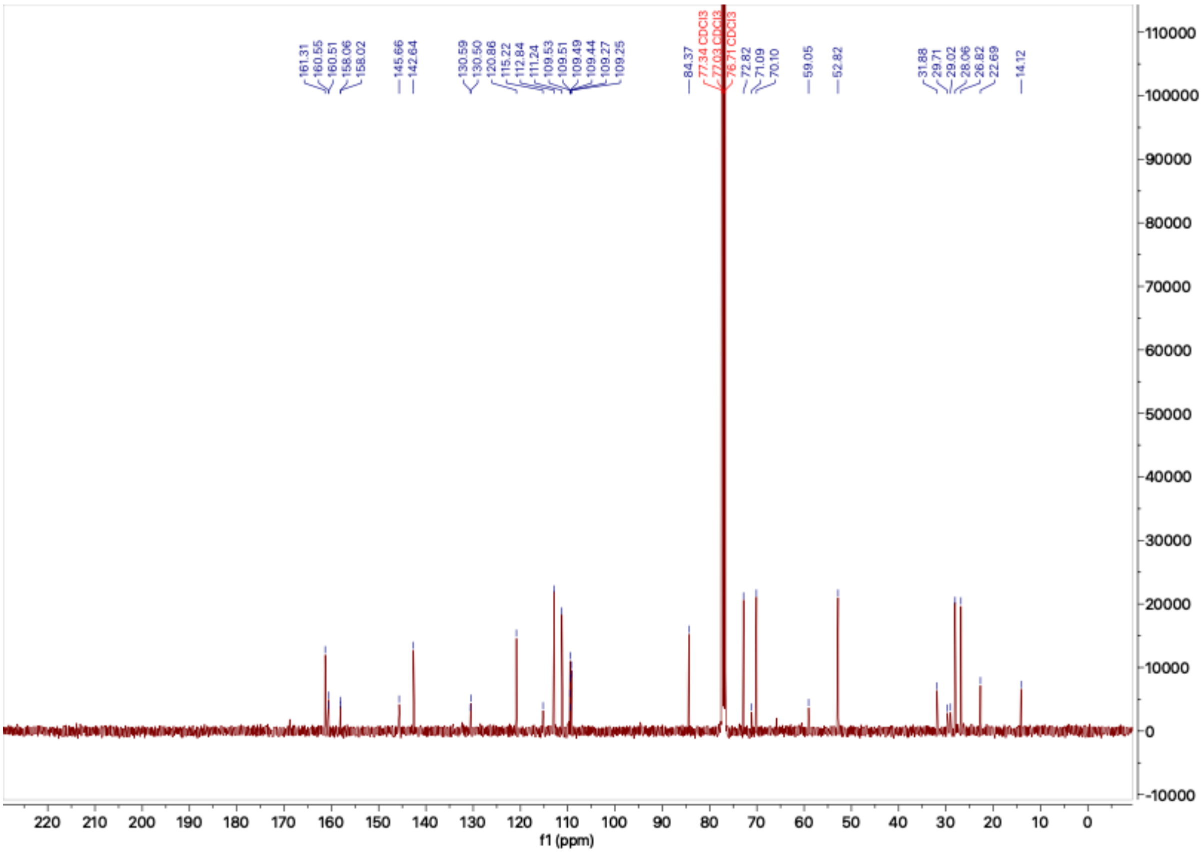

161.31
160.55
160.51
158.06
158.02
145.66
142.64
130.59
130.50
120.86
115.22
112.84
111.24
109.53
109.51
109.49
109.44
109.27
109.25
84.37
77.34 CDCl3
77.03 CDCl3
76.71 CDCl3
72.82
71.09
70.10
59.05
52.82
31.88
29.71
29.02
28.06
26.82
22.69
14.12
f1 (ppm)

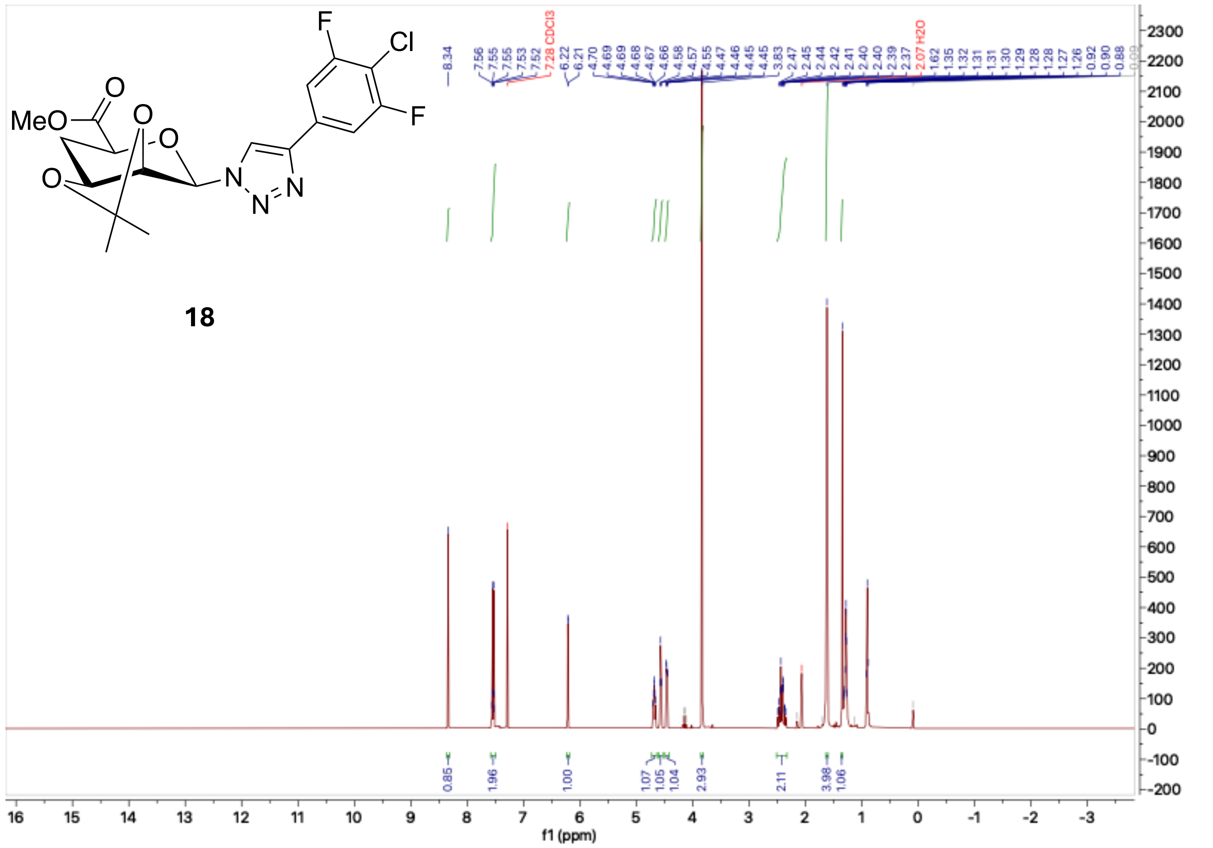

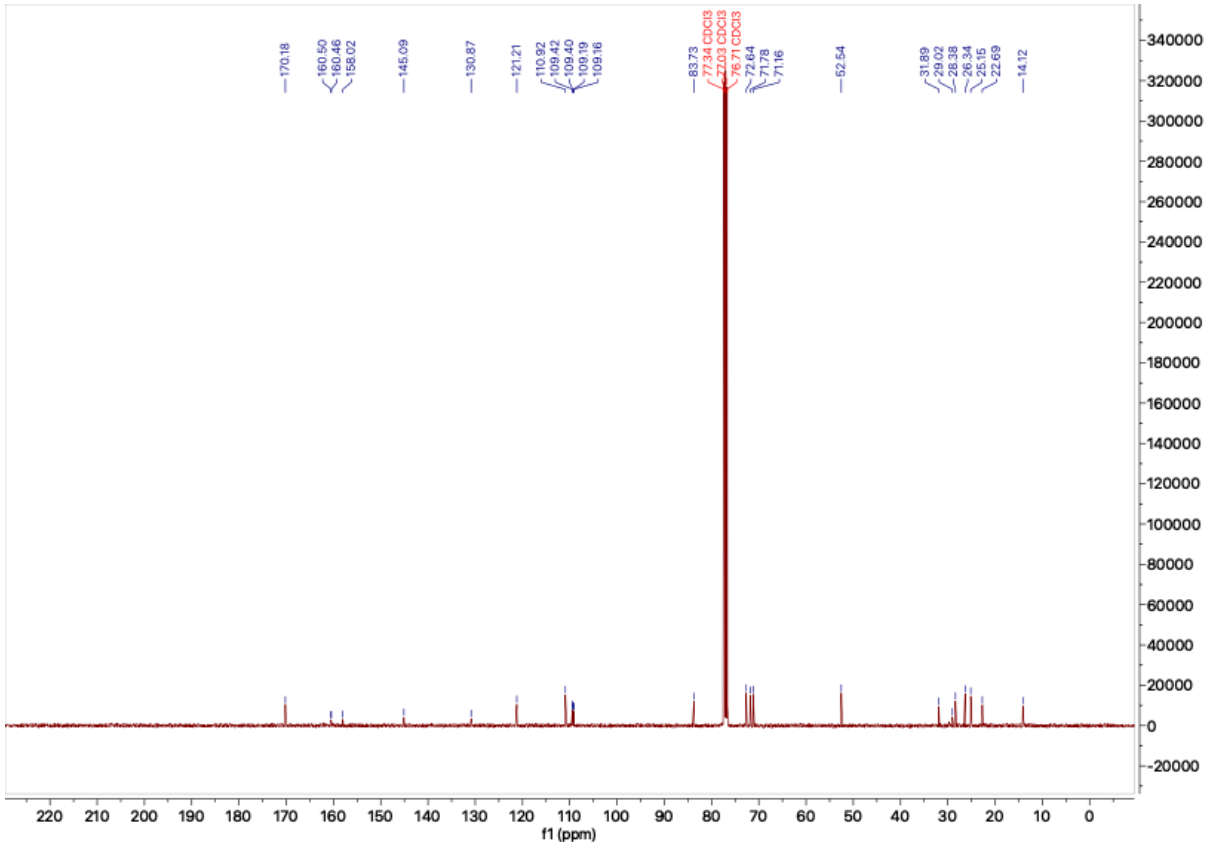

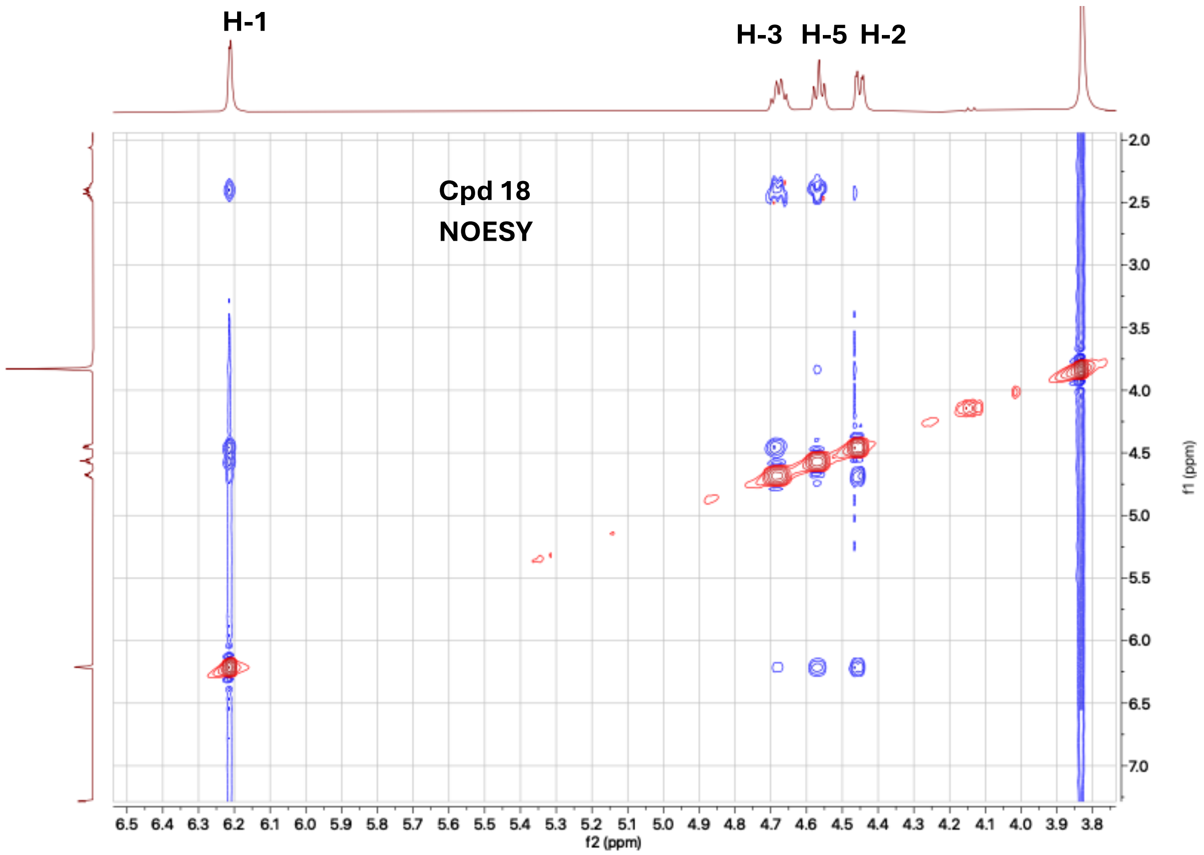
H-1
H-3 H-5 H-2
Cpd 18
NOESY
f2 (ppm)
f1 (ppm)

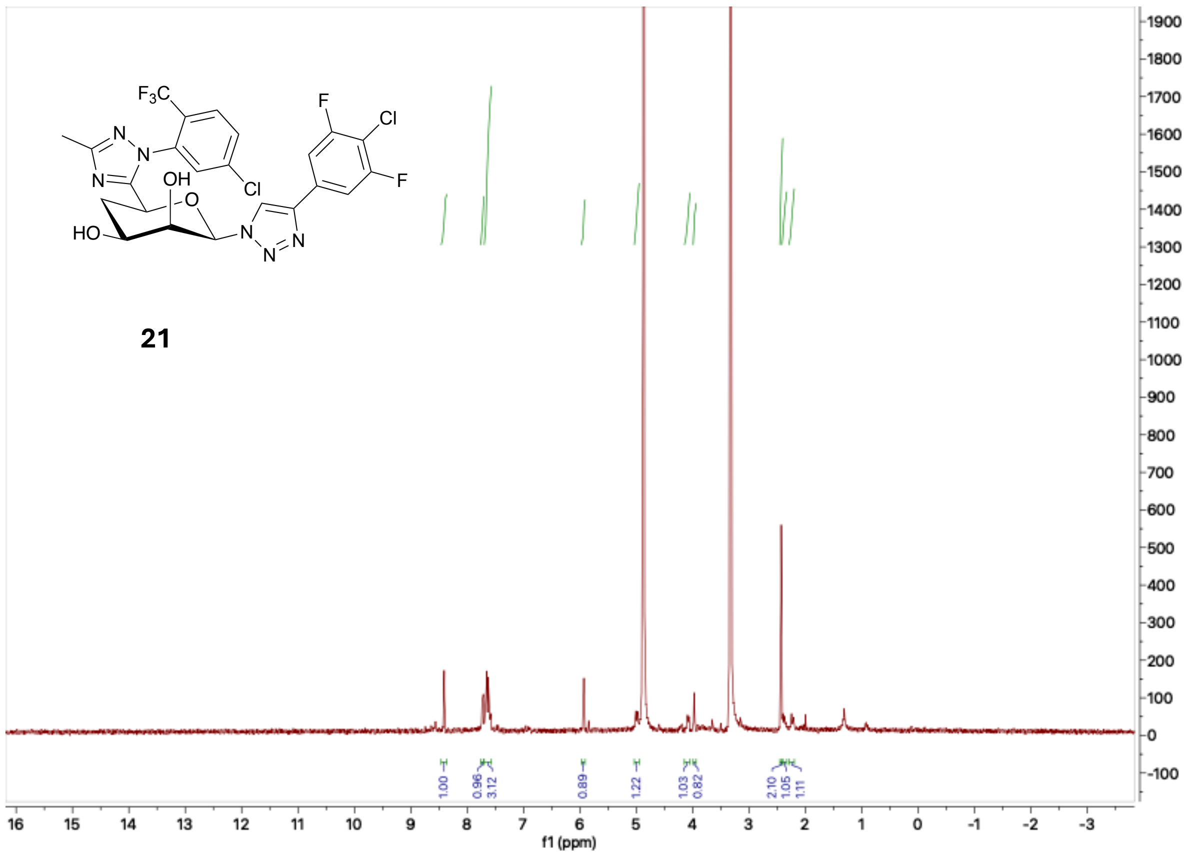
21
f1 (ppm)
1.00
0.96
3.12
0.89
1.22
1.03
0.82
2.10
1.05
1.11

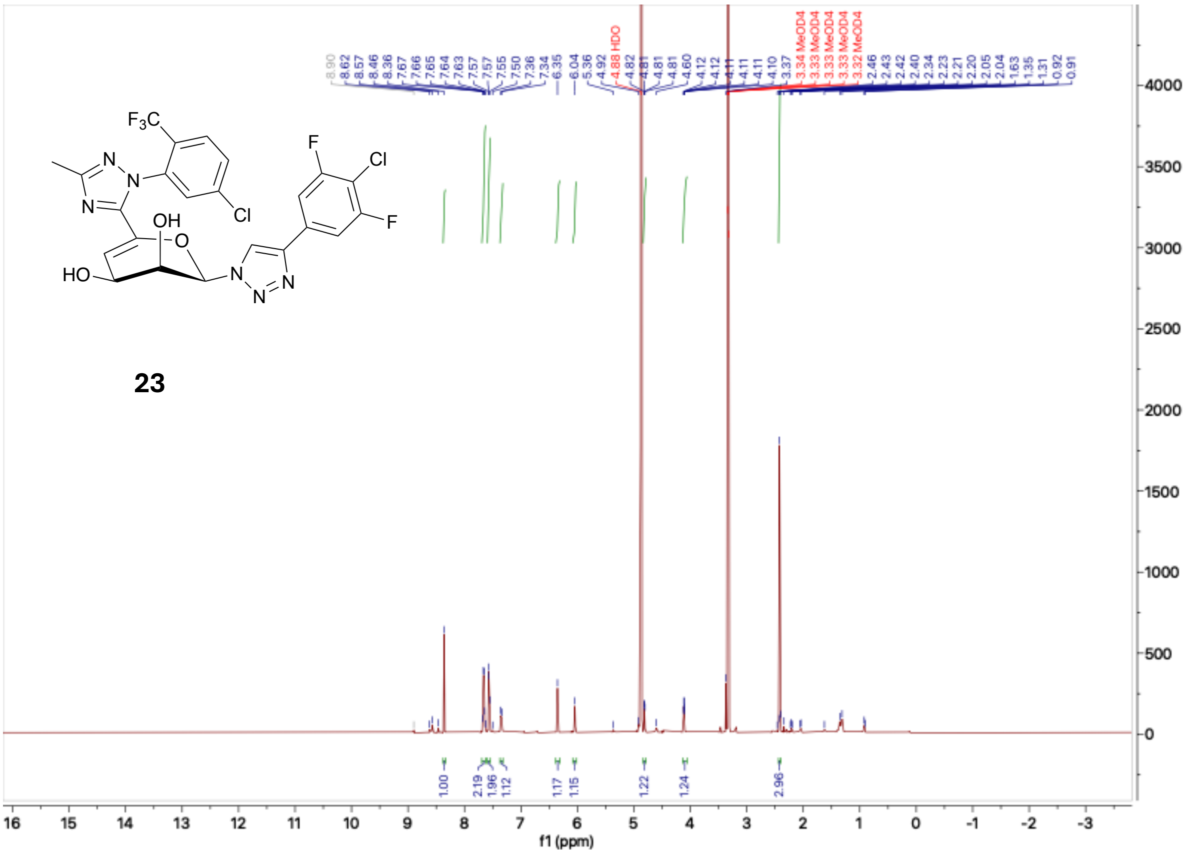

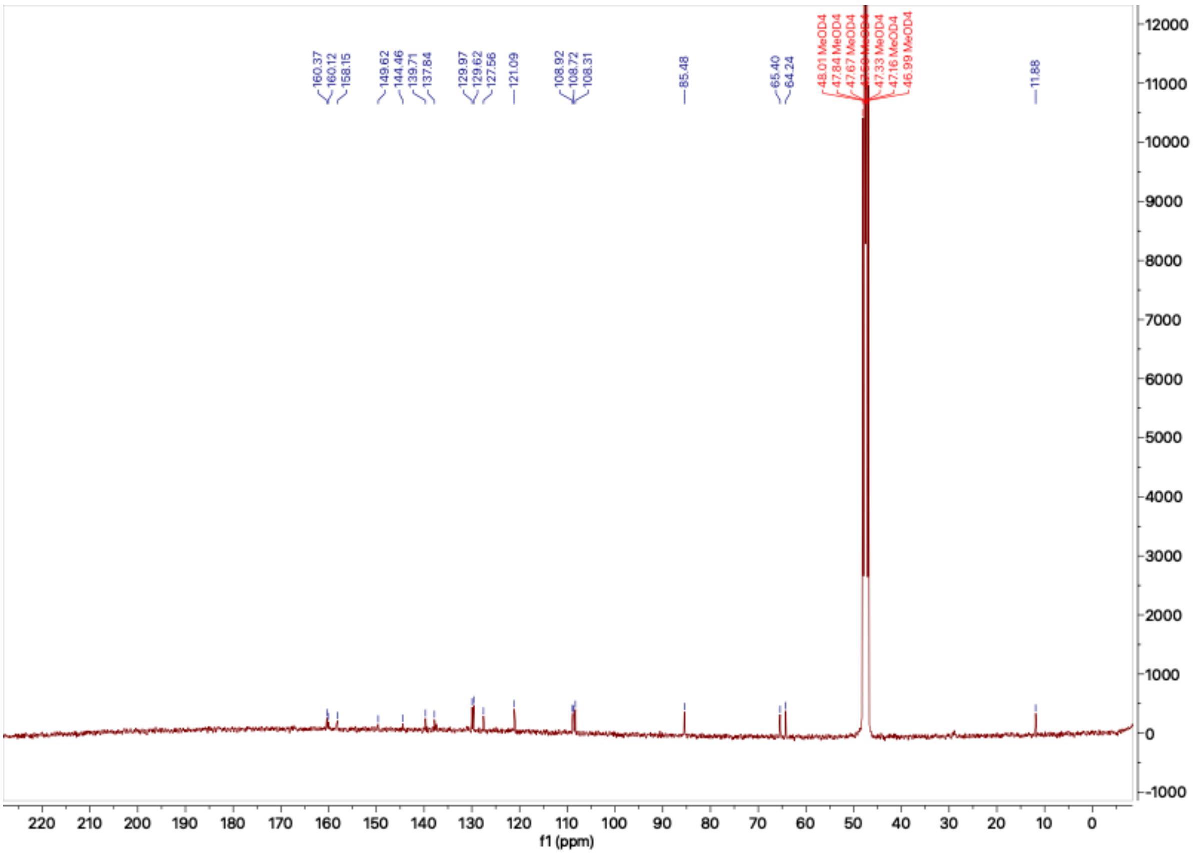

Supplement: Supplementary file 1 — Supplementary Material [file CBIC-27-e70319-s001.pdf]
